# Asprosin–PTPRD endocrine resistance links brain dysfunction and systemic wasting in Alzheimer’s disease

**DOI:** 10.21203/rs.3.rs-9972826/v1

**Published:** 2026-06-17

**Authors:** Bijoya Basu, Hesong Liu, Shrish Pandey, Hiba Obeid, Brennan Flannery, Yinghua Chen, Yuhyun Yun, Andrew Qian, Danica Parfyonov, Qianru Zhao, Hailan Liu, Jingzhi Meng, Gal Naveh, Santosh Hanumanthu, Jennifer Hoffman, Elizabeth Sabath Silva, Jim Leverenz, Lynn M. Bekris, C Dirk Keene, Juan C Bournat, Yanlin He, Ila Mishra, Andrew A. Pieper, Yong Xu, Atul R. Chopra

**Affiliations:** 1 Department of Genetics and Genome Sciences, Case Western Reserve University, Cleveland, OH, USA; 2 Department of Neuroscience and Physiology, College of Graduate Studies, Upstate Medical University, Syracuse, NY; 3 Harrington Discovery Institute, University Hospitals Cleveland Medical Center, Cleveland, OH, USA; 4 Department of Biomedical Engineering, Case Western Reserve University, Cleveland, OH, USA; 5 Protein Biophysics and Structure Biology Core Facility, Case Western Reserve University, Cleveland, OH, USA; 6 Pennington Biomedical Research Center, Louisiana State University, Baton Rouge, LA, 70808 USA; 7 Center for Molecular Psychiatry, University of South Florida, Morsani College of Medicine Tampa, FL, USA; 8 Lou Ruvo Center for Brain Health, Neurological Institute, Cleveland Clinic, Cleveland, OH, USA; 9 Genomic Medicine Institute Lerner Research Institute, Cleveland Clinic, Cleveland, OH, USA; 10 Department of Pathology, University of Washington School of Medicine, Seattle, WA, USA; 11 Department of Molecular and Cellular Biology, Baylor College of Medicine, Houston, TX, USA; 12 Division of Endocrinology, Diabetes and Metabolism, Department of Internal Medicine, College of Medicine, University of Kentucky, Lexington, KY, USA; 13 Department of Psychiatry, Case Western Reserve University School of Medicine, Cleveland, OH, USA; 14 Brain Health Medicines Center, Harrington Discovery Institute, University Hospitals Cleveland Medical Center, Cleveland, OH, USA; 15 Geriatric Psychiatry, GRECC, Louis Stokes VA Medical Center; Cleveland, OH, 44106, USA; 16 Institute for Transformative Molecular Medicine, School of Medicine, Case Western Reserve University; Cleveland, OH, 44106, USA; 17 Department of Neurosciences, Case Western Reserve University School of Medicine, Cleveland, OH, USA; 18 Department of Pathology, Case Western Reserve University School of Medicine, Cleveland, OH, USA; 19 Department of Medicine, University Hospitals Cleveland Medical Center, Cleveland, OH, USA

## Abstract

Alzheimer’s disease (AD) is characterized by cognitive decline and systemic frailty, but mechanisms linking brain dysfunction to organismal wasting remain unclear. We identify PTPRD, a human-genetically supported regulator of tau pathology, as a high-affinity receptor for amyloid-β (Aβ). Aβ competitively binds the PTPRD extracellular domain, opposing the endogenous ligand asprosin and producing a state of functional signaling insufficiency despite preserved receptor and ligand levels. Circuit-specific *Ptprd* deletion reveals anatomically distinct memory domains, while increasing asprosin in two AD mouse models restores Ptprd signaling and improves memory in a stage- and circuit-dependent manner. In advanced disease, asprosin supplementation substantially improves weight loss, muscle atrophy, strength, endurance and frailty. These findings support a model in which Aβ disrupts a CNS metabolic signaling axis linking cognition and systemic physiology and suggest that restoring ligand–receptor balance at PTPRD may ameliorate key features of AD.

## Introduction

Alzheimer’s disease (AD) is characterized by progressive cognitive decline accompanied, in many patients, by weight loss, sarcopenia, and increased frailty, all of which contribute to morbidity and mortality. While amyloid-β (Aβ) plaques and neurofibrillary tangles remain defining pathological hallmarks, how amyloid burden disrupts specific signaling pathways that coordinate brain function with systemic energy homeostasis and peripheral tissue maintenance is not fully understood^1–4^. However, emerging evidence indicates that Aβ may exert important effects not merely through neuronal toxicity, but also by acting as a pathogenic ligand that can subvert critical receptor signaling pathways^5–9^ in a manner reminiscent of endocrine resistance states in metabolic disease.

An intriguing candidate for such disruption is the receptor-type tyrosine phosphatase PTPRD, a receptor known to be activated by the orexigenic adipokine asprosin^10–13^ to modulate appetite, adiposity, and hydration through hypothalamic^12,14^ and cerebellar^15^ circuits. Human genetic studies place *PTPRD* at the intersection of multiple core features of AD biology, linking it not only to disease susceptibility but also to the rate of cognitive decline and, most strikingly, to neurofibrillary tangle (NFT) burden^16–20^. In a genome-wide analysis of NFT pathology across 909 prospective autopsies, *PTPRD* variation reached genome-wide significance (rs560380; p = 3.8 × 10^−^^8^) and explained ~3% of variance in tangle burden — an effect size second only to *APOE* across the entire genome^17^. Critically, this association was independent of amyloid plaque burden and persisted in individuals with zero neuritic plaques, suggesting that PTPRD may govern a mechanistically distinct, amyloid-independent pathway to tau pathology. This is consistent with a convergent model: loss-of-function *PTPRD* variants reduce receptor signaling constitutively and independently of amyloid, while in sporadic AD the same functional state may be acquired when Aβ accumulation functionally silences an otherwise intact receptor. In both cases, the pathological endpoint is reduced PTPRD signaling, with plausible downstream consequences for tau pathology. Beyond tangle burden, *PTPRD* has been linked to accelerated decline in memory and language, multimodal imaging-based AD classification, and late mild cognitive impairment, extending its genetic relevance from established dementia to prodromal disease^16,18–20^. Together, these convergent human genetic signals position PTPRD as a progression-relevant target embedded in the biology that drives tau pathology and neurodegeneration at least in part independently of amyloid burden. This human genetic signal is reinforced by cross-species experimental evidence indicating that reduced Ptprd function is sufficient to impair cognition and promote tau-relevant pathology. Global deletion of *Ptprd* has long been associated with learning and memory abnormalities^21^, and more recent work shows that Ptprd deficiency in aged mice increases pathological tau phosphorylation and impairs cognitive function^22^. Moreover, recent genetic and pharmacologic studies in 3xTg-AD mice indicate that reduced PTPRD function exacerbates AT8-positive tau pathology, whereas positive allosteric modulation of PTPRD phosphatase activity markedly suppresses tau pathology *in vivo*^23^, demonstrating bidirectional modulation of tau. Together, these observations support the view that PTPRD represents an important node for AD pathogenesis.

Against this backdrop, the possibility that Aβ could directly diminish PTPRD signaling by competing with its endogenous endocrine ligand asprosin offers a parsimonious explanation for how amyloid burden could engage a genetically and functionally validated pathway linking cognitive decline, circuit dysfunction, and downstream neurodegenerative progression. This hypothesis has translational implications: if key aspects of AD-related cognitive and systemic decline arise from a competitive reduction in signaling rather than from fixed loss of receptor expression, then at least some functional capacity may remain pharmacologically accessible even in advanced disease. Late-stage AD is frequently accompanied by weight loss, muscle wasting, and clinical frailty, features that contribute strongly to poor outcomes but are often viewed as secondary and largely irreversible consequences of neurodegeneration^24–28^. We considered the possibility that these manifestations reflect, in part, a failure of Ptprd-mediated endocrine signaling connecting systemic metabolic state to brain circuits that support memory and resilience. In this view, AD progression may give rise to an “Alzheimer’s endocrine resistance” state at the asprosin–Ptprd axis, analogous in concept to insulin resistance in type 2 diabetes, which might remain pharmacologically modifiable even in advanced disease.

By examining the interplay between Aβ and asprosin at the Ptprd interface, we tested the hypothesis that increasing the availability of asprosin can displace Aβ from the receptor, thereby unlocking suppressed signaling. Our findings demonstrate that Aβ can act as a high-affinity, pathological antagonist at Ptprd, and that increasing the availability of asprosin can restore Ptprd signaling, improve defined memory domains, and mitigate features of systemic wasting in AD mouse models. Together, these results support a model in which an AD-associated state of receptor-level endocrine resistance emerges at a CNS metabolic hub and provide a mechanistic link between amyloid burden, cognitive decline, and late-life frailty, raising the possibility that rebalancing ligand–receptor interactions at this axis could be therapeutically exploitable.

## Results

### Amyloid-β binds Ptprd with nanomolar affinity *in vitro* and forms a complex with Ptprd in AD-relevant brain regions

**Figure 1:**
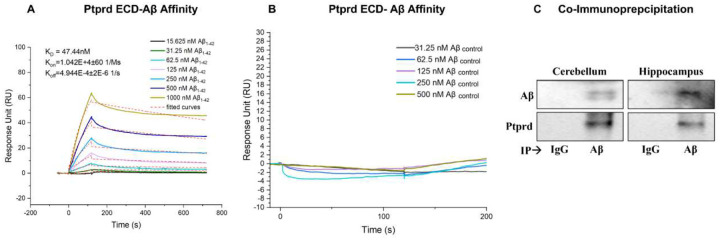
Amyloid-β binds Ptprd with nanomolar affinity *in vitro* and in critical brain regions of AD mice (A) Binding affinity between Aβ_1–42_ and the extracellular domain of Ptprd (Ptprd-ECD) quantified using Surface Plasmon Resonance (SPR). (B) Binding affinity between the reverse-sequence Aβ_42–1_ and Ptprd-ECD quantified using SPR. (C) Co-immunoprecipitation of endogenous β-amyloid (Aβ) from brain tissue lysates followed by immunoblot analysis for Ptprd (~120 kDa); β-amyloid is detected at ~30 kDa.

Genome-wide association studies consistently identify human genetic variation at the *PTPRD* locus as a significant risk factor for AD susceptibility, the rate of cognitive decline, and the density of neurofibrillary tangles^16–20^. To investigate the molecular basis of Ptprd dysfunction in AD, we performed surface plasmon resonance (SPR) using the Ptprd extracellular domain. We observed that Aβ oligomers bind directly to Ptprd with a dissociation constant (K_D_) of 47.44 nM, an affinity comparable to its endogenous ligand, asprosin^14^ (Fig. 1A). This interaction is specific, as reverse-sequence Aβ controls exhibited no binding (Fig. 1B). We further examined this interaction *in vivo* using co-immunoprecipitation (co-IP) from brain lysates of 5xFAD mice. Endogenous Aβ co-precipitated with the Ptprd receptor in both the cerebellum and hippocampus, sites of high *Ptprd* expression^29^ (Fig. 1C), suggesting that Aβ and Ptprd form a stable physical complex within the native brain environment under conditions relevant to AD.

### Aβ occupancy is associated with reduced Ptprd phosphatase signaling

**Figure 2:**
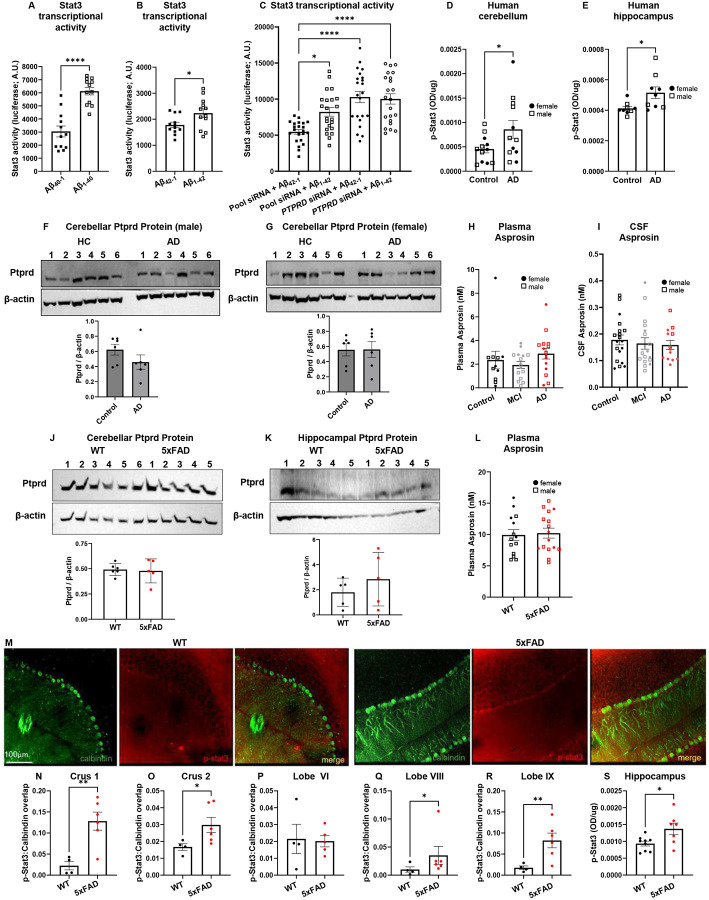
Aβ occupancy is associated with reduced Ptprd phosphatase signaling (A,B) STAT3-response element luciferase activity in HEK293T cells transfected with 4×M67 pTATA-TK-Luc, measured 24 h after 200 nM Aβ_1–40_ or Aβ_40–1_ (A), and Aβ_1–42_ or Aβ_42–1_ (B) (three technical replicates, 12 biological replicates/group). (C) STAT3-response element luciferase activity in HEK293T cells transfected with reporter and treated with control or Ptprd siRNA, measured 24 h after Aβ_1–42_ or Aβ_42–1_ (three technical replicates, 22 biological replicates/group). (D,E) p-Stat3 levels in age- and sex-matched human cerebellum (D; n = 7 healthy males, n = 7 AD males, n = 5 healthy females, n = 5 AD females) and hippocampus (E; n = 4 healthy males, n = 4 AD males, n = 5 healthy females, n = 5 AD females), normalized by protein concentration. Closed circles, females; open squares, males. (F,G) Representative western blots and quantification of β-actin and Ptprd in cerebellum of male (F) and female (G) AD donors vs age- and sex-matched controls. (H,I) Asprosin levels in human donors: plasma (H; n = 11 control, n = 15 MCI, n = 15 AD) and CSF (I; n = 19 control, n = 18 MCI, n = 13 AD). (J,K) Representative western blots and quantification of β-actin and Ptprd in cerebellar (J; n = 5–6/group) and hippocampal (K; n = 5/group) lysates from 9-month-old male 5xFAD vs WT mice. (L) Plasma asprosin in 6-month-old male and female 5xFAD mice (n = 16) vs WT controls (n = 14). (M) Representative images of anterior Crus 1 showing Calbindin (green) and p-Stat3 (red) in WT and 5xFAD mice. (N–R) Quantification of Purkinje neuron (Calbindin) and p-Stat3 overlap in cerebellar regions (n = 4–6/group). (S) Hippocampal p-Stat3 in male 9-month-old 5xFAD vs WT mice (7–9/group). Data are presented as mean ± SEM with individual data points shown in (A-E,N-S). One-way ANOVA (C, H, I); unpaired two-sided t-tests (A,B,D,E,F,G,J,K,L,S); one-sided t-tests (N–R) *p<0.05, **p<0.01, ***p<0.001, and ****p<0.0001.

We next examined how Aβ occupancy affects PTPRD function. In a HEK293T cell model expressing a Stat3-dependent luciferase reporter, exposure to recombinant Aβ species (both Aβ_1-_40 and Aβ_1–42_) significantly elevated STAT3 transcriptional activity (Fig. 2A and 2B). Given that PTPRD normally suppresses STAT3 activity through dephosphorylation^14,30^, this increase is consistent with a functional reduction in PTPRD-mediated phosphatase signaling. Consistent with that, *PTPRD* knockdown prevented Aβ-mediated increase in STAT3 transcriptional activity (Fig. 2C, Suppl. Fig. 1A).

This signaling deficit was mirrored *in vivo*: the cerebellum and hippocampus, both sites of high brain Ptprd expression^29^, of human AD donors showed markedly elevated levels of phosphor-STAT3 (p-STAT3) (Fig. 2D, 2E) despite unchanged total PTPRD protein abundance (Fig. 2F, 2G) and unchanged plasma and CSF asprosin levels (Fig. 2H, 2I). Similarly, in 5xFAD mice, cerebellar and hippocampal Ptprd protein expression (Fig. 2J, 2K) and plasma asprosin levels (Fig. 2L) remained unaltered concurrent with increased cerebellar Purkinje neuron (Fig. 2M–R) and hippocampal (Fig. 2S) p-Stat3. Together, these findings suggest that Aβ contributes to a state of functional PTPRD signaling insufficiency in the context of preserved receptor expression and normal ligand availability – a pattern compatible with receptor-level endocrine resistance.

### Reduced Ptprd activity is associated with loss of circuit-specific memory domains

**Figure 3:**
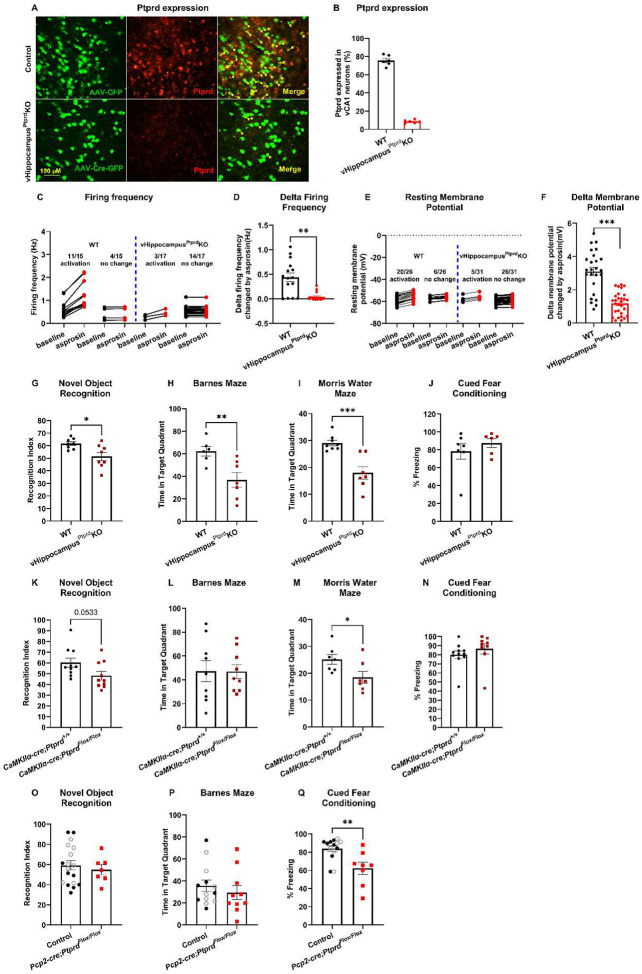
Reduced Ptprd activity results in the loss of circuit-specific memory domains (A,B) Representative immunostaining of AAV (green) and Ptprd (red) and Ptprd expression in ventral hippocampus following AAV-Cre injection in Ptprd^Flox/Flox^ mice. (C–F) Electrophysiology in ventral hippocampal neurons with or without asprosin: AP firing frequency (C), Δ firing frequency (D; n=15 WT, n=18 KO), resting membrane potential (E), and Δ membrane potential (F; n=26 WT, n=29 KO). (G–J) Behavioral assays in WT and age-matched vHippocampusPtprd KO mice: Novel Object Recognition (G; n=8/group), Barnes Maze (H; n=6 WT, n=7 KO), Morris Water Maze (I; n=8/group), and Cued Fear Conditioning (J; n=6 WT, n=7 KO). (K–N) Behavioral assays in 10–12-week-old male CaMKII-cre;Ptprd^Flox/Flox^ mice vs age- and sex-matched CaMKII-cre controls: Novel Object Recognition (K; n=10/group), Barnes Maze (L; n=9/group), Morris Water Maze (M; n=7/group), and Cued Fear Conditioning (N; n=9 KO, n=11 control). (O–Q) Behavioral assays in 12-week-old male Pcp2-cre;Ptprd^Flox/Flox^ mice vs age- and sex-matched Pcp2-cre and Ptprd^Flox/Flox^ controls: Novel Object Recognition (O; n=7 KO, n=9, n=9 controls), and Barnes Maze (P; n=10 KO, n=6, n=7 controls), Cued Fear Conditioning (Q; n=8 KO, n=7, n=7 controls). Data are represented as mean ± SEM with individual data points (B,D,F,G-Q) or individual points (C,E). Two-sided unpaired (D,F) or paired (C,E) t-tests. Statistical significance is indicated as *p<0.05, **p<0.01, ***p<0.001, and ****p<0.0001.

Human genetic and mouse deletion studies converge on *PTPRD* as a determinant of cognitive function^16–20^. To determine the brain regions underlying these deficits, we utilized circuit-specific knockout (KO) models. Deletion of *Ptprd* specifically from ventral hippocampal circuits via stereotaxic delivery of Cre-recombinase (Fig. 3A, 3B), resulted in significantly reduced baseline neuronal activity measured via slice electrophysiology (Fig. 3D, 3F), and notably, an inability of recombinant asprosin to increase excitability (Fig. 3C, 3E). Behaviorally, we observed robust impairments in recognition (Fig. 3G) and spatial memory (Fig. 3H, 3I, Suppl. Fig. 2A, 2B), while cued associative learning was spared (Fig. 3J, Suppl. Fig. 2C). This adult-restricted deletion approach obviated developmental confounds, and knockout mice showed no locomotor deficits (Suppl. Fig. 2E–F).

Corroborating these findings, genetic *CaMKIIα-Cre*–mediated deletion of *Ptprd* from forebrain excitatory neurons recapitulated the spatial and recognition memory deficits (Fig. 3K–N, Suppl. Fig. 2G–I). Spatial impairment was detected in the Morris Water Maze but not Barnes Maze, likely reflecting two complementary factors: first, the *CaMKII-Cre* strategy targets only excitatory neurons, producing a functionally smaller and more incomplete hippocampal circuit disruption than the broader stereotaxic approach^31^; and second, Barnes Maze, unlike MWM, can be solved using serial non-spatial search strategies that do not obligate hippocampal allocentric mapping^32^, potentially masking subtle deficits in partially disrupted circuits. No effect was noted on body weight (Suppl. Fig. 2K) or locomotion (Suppl. Fig. 2L, 2M), ruling out metabolic or motor confounds. Short working memory, assessed by Y-maze, remained intact in both hippocampal deletion models (Suppl. Fig. 2D, 2J).

Conversely, ablation of *Ptprd* from cerebellar Purkinje neurons – a known asprosin-Ptprd–activated population^15^ with established links to associative memory^33–35^ – selectively impaired cued associative memory (Fig. 3Q, Suppl. Fig. 3E, 3L, 3M) while leaving recognition (Fig. 3O, Suppl. Fig. 3I) and spatial memory intact (Fig. 3P, Suppl. Fig. 3D, 3J, 3K). This double dissociation indicates that Ptprd supports anatomically segregated memory circuits: cued associative memory via cerebellar Purkinje neurons and recognition/spatial memory via ventral hippocampal circuits. As with hippocampal and forebrain excitatory neuron deletions, Y-maze working memory was unaffected (Suppl. Fig. 3C, 3H), and no locomotor or anxiety abnormalities were observed (Suppl. Fig. 3A, 3B, 3F, 3G), excluding motor or emotional confounds as alternative explanations for the fear-conditioning deficit. Control comparisons between mice carrying *floxed Ptprd* alleles alone and those expressing *Pcp2-Cre* alone confirmed that neither element independently altered behavior or cognition (Suppl. Fig. 3N–Y).

To confirm circuit specificity, deletion of *Ptprd* from hypothalamic AgRP neurons – the primary substrate for Ptprd′s orexigenic function^11,13–15,36^ – produced no cognitive deficits (Suppl. Fig. 4AE), demonstrating that Ptprd′s role in memory is circuit-selective rather than a general property of all Ptprd-expressing neurons. Taken together, these results indicate that Ptprd signaling is critically involved in recognition, spatial, and associative memory through anatomically and functionally segregated neural circuits.

### Asprosin reduces Aβ occupancy at Ptprd and restores receptor signaling

**Figure 4:**
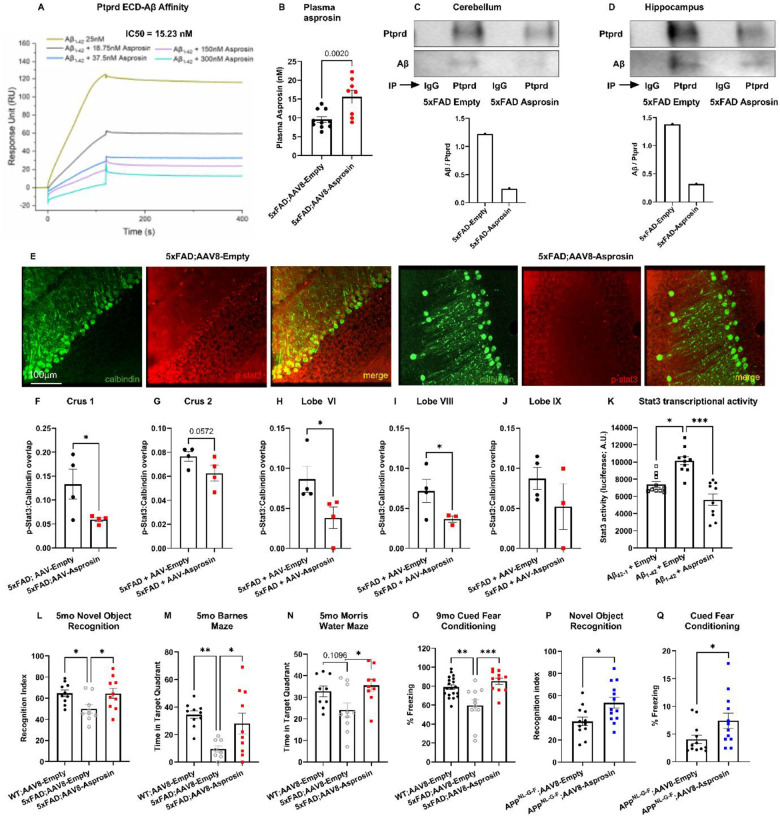
Asprosin reduces Aβ occupancy at Ptprd and restores receptor signaling (A) Competitive binding of asprosin and Aβ_1–42_ to Ptprd-ECD measured by SPR. (B) Plasma asprosin levels in 5xFAD mice (AAV8-Empty, n = 10 or AAV8-Asprosin, n = 8). (C,D) Immunoprecipitation of Ptprd (~120 kDa) from cerebellar (C) and hippocampal (D) lysates followed by immunoblotting for endogenous Aβ; Aβ detected at ~30 kDa in 9-month-old 5xFAD mice treated with AAV-Asprosin or AAV-Empty. (E) Representative anterior Crus 1 images showing Purkinje neurons (Calbindin, green) and p-Stat3 (red). (F–J) Quantification of Purkinje neuron and p-Stat3 overlap in associative cerebellar regions (n = 3–4/group). (K) STAT3 luciferase activity in HEK293T cells transfected with reporter and empty or IL2-his-asprosin plasmid, treated with Aβ_1–42_ or Aβ_42–1_ (200 nM); measured 6 h after treatment (three technical replicates, 10 biological replicates/group). (L–N) Memory in 5-month-old males: WT (AAV8-Empty) and 5xFAD mice (AAV8-Empty or AAV8-Asprosin; 1×1012 GC/mouse) assessed by Novel Object Recognition (L), Barnes Maze (M), and Morris Water Maze (N). (O) Associative memory in 9-month-old males (n = 19 WT; n = 11 per 5xFAD group). (P,Q) Recognition (P; n=12, n=13) and associative memory (Q; n=12/group) in APP^NL-G-F^ mice. Data are mean ± SEM with individual points. One-way ANOVA (K–O); one-sided (B, F–J) or two-sided (P,Q) unpaired t-tests. *p<0.05, **p<0.01, ***p<0.001, ****p<*0.0001*. Binding analyzed using BIAevaluation.

We hypothesized that the hormone asprosin, the homeostatic ligand of Ptprd, could functionally oppose the pathological ligand Aβ at this receptor. SPR competition assays demonstrated that asprosin effectively outcompetes Aβ for Ptprd binding in a dose-dependent manner, with an IC_50_ of 15.23 nM (Fig. 4A). *In vivo*, sustained asprosin supplementation in 5xFAD mice was achieved using AAV-asprosin, an approach previously employed to elevate circulating asprosin and drive central effects — including appetite^11,14,36,37^ and thirst^15^ induction — via asprosin′s well-documented capacity to cross the blood-brain barrier^13^ and act on hypothalamic^11,14,36^ and cerebellar^15^ Ptprd circuits. Hepatic AAV-asprosin expression increased plasma asprosin levels (Fig. 4B), and this peripherally-derived elevation was sufficient to reduce Aβ occupancy of Ptprd in both cerebellum and hippocampus (Fig. 4C, 4D). Notably, this anti-Ptprd pulldown reciprocally validates the Aβ-Ptprd physical interaction first established by anti-Aβ co-IP in Fig. 1C. This was accompanied by restored Ptprd phosphatase signaling, evidenced by decreased p-Stat3 levels in Purkinje neurons (Fig. 4E–J). Similarly, in our cell-based signaling model, asprosin exposure reversed Aβ-induced STAT3 transcriptional activity (Fig. 4K, Suppl. Fig. 4O), supporting a receptor-level competitive mechanism rather than non-specific cytotoxicity.

### Asprosin supplementation improves Ptprd-linked memory domains in two AD models

Consistent with the restoration of signaling, asprosin treatment led to the recovery of memory performance across different stages of disease. In 5-month-old 5xFAD mice, asprosin treatment resulted in significant improvement of recognition (Fig. 4L) and spatial memory (Fig. 4M, 4N, Supp. Fig. 4I, 4J). Cued associative memory was found to be intact in 5xFAD mice at this age, and asprosin supplementation did not enhance this memory domain over baseline (Suppl. Fig. 4G, 4H). Y-maze measured short-working memory was also found to be intact in 5xFAD mice at this age, and asprosin supplementation did not enhance this memory domain over baseline either (Suppl. Fig. 4F). Swim speed and distance during MWM were equivalent between groups (Suppl. Fig. 4M, 4N), confirming that the spatial memory deficit was not attributable to impaired swimming ability.

In 9-month-old 5xFAD mice, a stage commonly associated with substantial neurodegeneration^38–40^, sustained asprosin supplementation robustly rescued cued associative memory (Fig. 4O, Suppl. Fig. 5A, F–G), while not affecting recognition, spatial or short-working domains (Suppl. Fig. 5B–D, 5H–J) or locomotion (Suppl. Fig. 5E,K). This selective rescue likely reflects the anatomical preservation of the cerebellum in advanced AD, in contrast to the profound structural decay observed in the hippocampus^1,41–43^. These results suggest that even advanced AD can retain Ptprd-dependent memory circuits that remain responsive to endocrine modulation.

### Independent experiments in the APP^NL-G-F^ model support asprosin efficacy across AD-relevant genetic backgrounds

To ensure the robustness and generalizability of our findings, the results were reproduced in an independent laboratory using the APP^NL-G-F^ knock-in model. This model avoids the non-physiological protein overexpression artifacts characteristic of the 5xFAD model, instead driving amyloid pathology under endogenous genetic control to better mirror human disease progression^40,44^. At 9 months, APP^NL-G-F^ mice exhibit moderate-to-advanced pathology with comparatively less hippocampal neurodegeneration than age-matched 5xFAD mice^39,40,44^. Consistent with this, asprosin supplementation significantly improved recognition (Fig. 4P) and cued associative memory (Fig. 4Q, Suppl. Fig. 5L), while spatial memory remained unaffected (Suppl. Fig. 5M) – an outcome consistent with the greater hippocampal tissue requirement of spatial relative to recognition memory^45^. No effect was noted on short-term working memory (Suppl. Fig. 5N) or locomotion (Suppl. Fig. 5O). This successful cross-model replication supports the asprosin–Ptprd axis as a promising, genetically anchored target across varying AD-relevant backgrounds.

### Asprosin markedly improves AD-associated cachexia and sarcopenia in advanced disease

**Figure 5:**
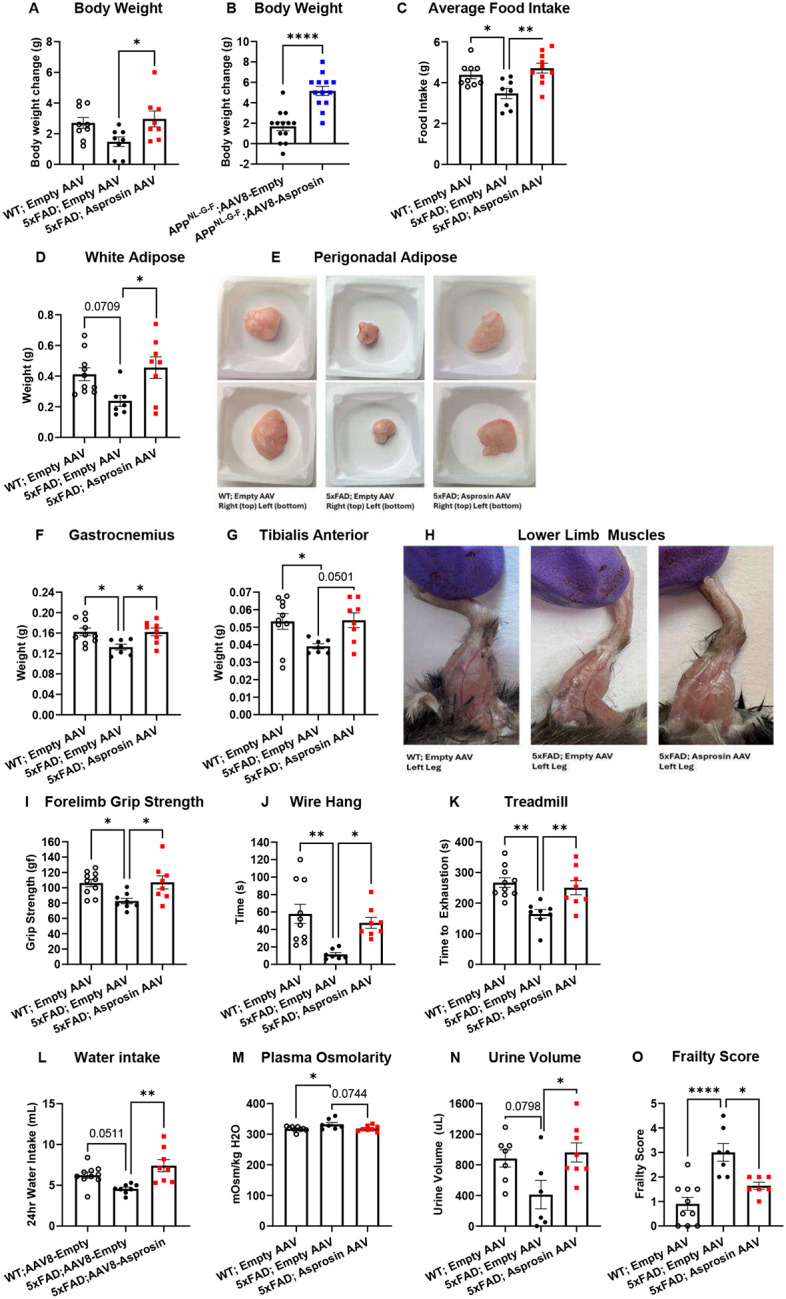
Asprosin markedly improves systemic cachexia and sarcopenia in advanced AD (A) Body weight change from 6 to 12 months in WT;AAV8-Empty (n=9), 5xFAD;AAV8-Empty (n=8), and 5xFAD;AAV8-Asprosin (n=8) mice. (B) Body weight in APP^NL-G-F^;AAV8-Empty (n=13) and APP^NL-G-F^;AAV8-Asprosin (n=13). (C) 24-hour food intake in 12-month-old WT;AAV8-Empty (n=10), 5xFAD;AAV8-Empty (n=8), and 5xFAD;AAV8-Asprosin (n=8). (D,E) Perigonadal fat weight (D; n=10, 7, 8) and representative images (E). (F–H) Gastrocnemius (F; n=10, 7, 8) and tibialis anterior (G; n=10, 7, 8) muscle weights with representative images (H). (I–K) Functional measures: forelimb grip strength (I; n=10, 8, 8), wire hang (J; n=10, 8, 8), and treadmill endurance (K; n=10, 7, 8). (L–N) Hydration and metabolic measures: water intake (L; n=10, 8, 8), plasma osmolality (M; n=9, 7, 8), and urine volume (N; n=7, 6, 8). (O) Frailty score (n=10, 7, 7). Data are mean ± SEM with individual points. One-way ANOVA with Tukey post hoc tests (A,C,D,F,G,I–O) or two-sided t-test (B). *p<0.05, **p<0.01, ***p<0.001, ****p<*0.0001*.

In highly advanced AD (12-month-old 5xFAD mice), the disease progresses to a lethal systemic state characterized by cachexia and sarcopenia, and rivals advanced cancers in mortality^38,46,47^. These mice exhibit profound fat and muscle wasting, reduced food intake, and severe frailty, reflecting a disease stage widely considered irreversible^38,46,47^. Given the established food intake, adiposity, and hydration modulating functions of the asprosin-Ptprd axis via CNS circuits^11,13–15,36^, we investigated the effects of asprosin supplementation on AD-induced cachexia. Notably, chronic asprosin supplementation markedly improved these features: treated 12-month-old 5xFAD mice showed significant increases in body weight (Fig. 5A) (independently replicated in 9-month-old APP^NL-G-F^ mice, Fig. 5B), food intake (Fig. 5C), adipose mass (Fig. 5D and 5E), and fast-twitch hindlimb muscle mass (Fig. 5F, 5G, 5H), and exhibited functional performance in grip strength (Fig. 5I), wire-hang (Fig. 5J), and treadmill endurance (Fig. 5K) that approached WT levels. In contrast, the slow-twitch soleus muscle was spared from AD-associated atrophy and showed no significant change upon asprosin treatment (Suppl. Fig. 5P), suggesting that asprosin selectively counteracts pathological wasting rather than inducing non-specific muscle hypertrophy. Additionally, asprosin brought hydration measures closer to WT levels (Fig. 5L, 5M and 5N) and significantly improved systemic frailty measured via a 31-point composite assay (Fig. 5O), consistent with the interpretation that systemic deterioration in this AD model reflects, at least in part, an endocrine resistance state that can be mitigated by restoring asprosin-Ptprd signaling.

## Discussion

We find that amyloid-β can function as a high-affinity competitive antagonist of the Ptprd receptor, uncoupling memory and metabolic circuits from the fasting-induced hormone asprosin. This competitive blockade produces a state of receptor-level endocrine resistance despite preserved Ptprd abundance and normal asprosin levels, redefining the amyloid milieu from a purely toxic environment to one in which pathological ligands outcompete homeostatic endocrine signals. Within this framework, PTPRD emerges as a CNS metabolic hub that integrates systemic nutritional state with discrete memory circuits, and Aβ-driven endocrine resistance at this node provides a simple mechanism to couple cognitive decline to late-life weight loss, sarcopenia, and frailty. By restoring signaling through the asprosin–Ptprd axis, we rescue Ptprd-dependent memory domains in a stage- and circuit-specific manner and reverse advanced AD-associated cachexia, muscle wasting, and frailty, indicating that key features of both brain failure and systemic metabolic collapse in AD remain pharmacologically modifiable.

Our findings suggest that the susceptibility of different memory domains to AD is governed by the selective vulnerability of anatomically segregated, Ptprd-dependent circuits. We show that while Ptprd signaling in hippocampal neurons is needed for spatial and recognition memory, its function in cerebellar Purkinje neurons specifically supports cued associative learning. This circuit-level specialization explains the stage-dependent pattern of cognitive rescue. Hippocampal circuits, which exhibit early synaptic and metabolic collapse^2,48–50^, lose the ability to respond to asprosin supplementation as the disease progresses beyond a certain threshold. In contrast, the relative anatomical/pathological sparing of the cerebellum^1,41–43,51,52^ – whose Ptprd-dependent signaling is suppressed but whose neuronal architecture remains structurally intact – provides a unique therapeutic window, allowing asprosin supplementation to improve cued associative memory even at advanced stages. This selective rescue suggests that the AD brain remains a mosaic of functional competence and suppressed potential, where behavioral recovery is dictated by the stoichiometric balance of pathological and homeostatic ligands within a preserved circuit.

A major concern with the 5xFAD model is that older mice often fail memory tests simply because they are too weak to move or swim^38,39,46,47^. Importantly, locomotion was unimpaired across all experimental models in this study, including every *Ptprd* circuit-specific knockout line and both AAV-asprosin treatment cohorts (Suppl. Figs. 2E–F, 2L–M, 3A, 3F, 4K–N, 5E, 5K, 5O), establishing the absence of motor confounds throughout. Beyond locomotion, in 5xFAD mice treated at 6-months-old, the treatment substantially restored muscle mass, strength, endurance and body weight, as measured at 12-months of age. Despite having normal muscular performance, mice treated at 6-months-old still failed the NOR and spatial memory tests, as measured at 9-months of age (Suppl. Figs. 5B–D, 5H–J) — with swim speed and distance during MWM confirming intact locomotor capacity during the task itself (Suppl. Fig. 4M, 4N). This suggests that their memory deficits exist independent of potential motor issues. These same mice also showed a full recovery in cued fear memory. However, because the mice showed no improvement in NOR or spatial exploration, where reduced hyperactivity would typically increase performance scores, the recovery in the fear conditioning task likely represents a genuine cognitive response to the cue.

At the molecular level, the discovery that Aβ binds the Ptprd extracellular domain with nanomolar affinity, virtually identical to that of its homeostatic ligand^14^, redefines the amyloid milieu. Rather than acting as a non-specific toxin, Aβ functions as a high-affinity competitive antagonist of the asprosin-Ptprd axis. This identifies a receptor-level bottleneck where pathological ligands outcompete homeostatic ones. Aβ has previously been shown to competitively bind the insulin receptor and interfere with insulin signaling^53^, providing precedent for Aβ acting as a pathological ligand that subverts normal hormone–receptor interactions leading to physiological deficits. Crucially, this state is imposed by extracellular ligand competition rather than receptor downregulation, leaving the downstream phosphatase machinery signal-competent and pharmacologically recoverable. From a translational perspective, the fact that Ptprd protein abundance and circulating asprosin levels remain preserved while signaling fails suggests that the Aβ-asprosin-Ptprd interface constitutes a discrete, druggable node. Our data support the concept that shifting the competitive equilibrium at this receptor by increasing the effective availability of the homeostatic ligand can restore signaling and improve both cognitive and systemic outcomes in AD models, without directly targeting Aβ burden.

This mechanism provides a molecular bridge to the robust genetic association between the *PTPRD* locus and tau pathology^17^. That bridge is further strengthened by independent evidence outside the present study. Human genetics place *PTPRD* near clinically meaningful AD biology, including cognitive decline and neurofibrillary tangle burden^16,17^, while prior mouse studies show that Ptprd deficiency promotes pathological tau phosphorylation and cognitive impairment^22^. Recent work in 3xTg-AD models extends this framework by showing that reduced PTPRD function worsens AT8-positive tau pathology, whereas positive allosteric modulation of PTPRD can markedly suppress it *in vivo*^23^. Importantly, we did not directly quantify tau species or neurofibrillary tangles in the present work; our experiments were instead designed to define how Aβ competitively blocks the asprosin–Ptprd axis and how restoring Ptprd signaling impacts circuit-specific memory domains and systemic wasting. Determining whether endocrine restoration at Ptprd is sufficient to modify tau pathology *in vivo* will require a dedicated set of experiments and is the focus of ongoing work that is conceptually distinct from the goals of the current study. Taken together with prior genetic and experimental evidence linking *PTPRD* to tau pathology, our findings motivate the hypothesis that amyloid can act as a pathological ligand that functionally silences a genetically and mechanistically tau-relevant pathway.

The implications of this axis extend beyond the CNS to the profound systemic deterioration that defines the clinical reality of AD^24,26–28,54,55^. While the restoration of food intake, adiposity, and hydration aligns with known CNS-mediated functions of the asprosin-Ptprd axis^11,13–15,36^, the mechanism underlying the rescue of skeletal muscle mass remains to be elucidated. It is possible that the reversal of sarcopenia is a downstream consequence of corrected central energy homeostasis; however, direct peripheral action of asprosin on muscle, the neuromuscular junction, or motor neuron-specific Ptprd signaling cannot be ruled out. Regardless of the specific site of action, the robust recovery of lean mass in geriatric AD mice demonstrates that the physical collapse associated with advanced AD is not merely a terminal decline, but reflects a state of active biological suppression that remains pharmacologically recoverable. Although our genetic and signaling data indicate that Ptprd is a principal receptor for asprosin in the CNS, the current *in vivo* rescue experiments do not formally exclude Ptprd-independent mechanisms of asprosin action. Future studies using conditional *Ptprd* deletion in the context of AAV-asprosin will be required to define the extent to which the cognitive and systemic benefits of asprosin are strictly Ptprd-dependent.

Collectively, these findings support the existence of an “Alzheimer’s endocrine resistance” state at the asprosin–Ptprd axis. In this state, a pathogenic ligand, Aβ, occupies a CNS metabolic receptor with nanomolar affinity, functionally insulating it from its homeostatic hormone despite preserved receptor and ligand abundance. This mechanism is conceptually analogous to endocrine resistance in classic metabolic diseases such as type 2 diabetes, but here it operates at a receptor that links systemic energy balance, circuit-specific memory functions, and late-life physical resilience. In addition to the established framework in which Aβ exerts direct neurotoxicity and promotes tau pathology, this endocrine resistance state provides a complementary route by which amyloid burden can drive both neuronal dysfunction and systemic decline. By shifting the competitive equilibrium at Ptprd in favor of asprosin, we show that this endocrine resistance state is pharmacologically modifiable, with coordinated improvements in defined memory domains and in cachexia, sarcopenia, and frailty. This positions the asprosin–Ptprd axis as a genetically anchored, druggable node through which AD pathology can be understood – and potentially treated – as a disorder of brain-centered metabolism and endocrine signaling.

## Methods

### Mouse Models

WT C57BL/6 mice (WT mice; Jackson Laboratory, JAX #:000664), *AgRP-IRES-cre* (C57BL/6-Agrptm1(cre)Lowl, Jackson Laboratory JAX #: 012899), *Pcp2-cre* (B6.129-Tg(Pcp2-Cre)2Mpin/j, Jackson Laboratory, JAX #:004146) and *CaMKIIα*-cre (B6.Cg-Tg(Camk2a-cre)T29-1Stl/J, Jackson Laboratory, JAX #: 005359) were purchased from Jackson Laboratories. Alzheimer’s mouse models 5xFAD mice (B6.Cg-Tg(APPSwFlLon,PSEN1*M146L*L286V) 6799Vas/Mmjax, Jackson Laboratory, JAX #034848-JAX) were purchased from Jackson Laboratories and App^NL-G-F^ mice were obtained from RIKEN, Japan(40).

Homozygous conditionally ready *Ptprd* floxed mice (Ptprd tm2c(KOMP)Wtsi) were mated with *AgRP-IRES-cre* (C57BL/6-Agrptm1(cre)Lowl) to create AgRP neuron specific knock-out of *Ptprd*. Homozygous conditionally ready *Ptprd* floxed mice (Ptprd tm2c(KOMP)Wtsi) were mated with *Pcp2-cre* (B6.129-Tg(Pcp2-Cre)2Mpin/j) to create Purkinje neuron specific knock-out of *Ptprd*. Homozygous conditionally ready *Ptprd* floxed mice (Ptprd tm2c(KOMP)Wtsi) were mated with *CaMKIIα*-cre (B6.Cg-Tg(Camk2a-cre)T29-1Stl/J) to create CaMKIIα-neuron specific knock-out of *Ptprd*.

5xFAD mice (B6.Cg-Tg(APPSwFlLon,PSEN1*M146L*L286V) 6799Vas/Mmjax, Jackson Laboratory, JAX #034848-JAX) were also bred with Purkinje neuron specific knock-out of *Ptprd* to create a line where *Ptprd* was constitutively knocked out of Purkinje neurons in the 5xFAD model.

Mice were housed in micro ventilators on a 12-hour light cycle (6am-6pm) in an animal facility maintained at 20–25°C and 40–60% humidity. Mice had ad libitum access to water and normal chow. Animal housing, husbandry, experiments, and euthanasia were conducted under animal protocols approved by the Case Western Reserve University Institutional Animal Care and Use Committee (protocol# 2018–0042). General health of mice was monitored by the CWRU animal resource center.

### Human Donor Protein Samples

Human donor samples from the University of Washington were provided from the following studies: Adult Changes in Thought (ACT) (5U19 AG066567) and Biological heterogeneity in ADRD (P30 AG066509). Snap-frozen cerebellar and hippocampal tissue was homogenized using N-PER Neuronal Protein Extraction Reagent (Thermo Fisher Scientific, 87792) supplemented with protease inhibitor (Thermo Fisher Scientific, 78429) and phosphatase inhibitor (Thermo Fisher Scientific, 78420) to produce protein lysates. Protein concentrations were quantified using the Pierce BCA Protein Assay Kit (Thermo Fisher Scientific, 23227).

### Human Biofluid Samples

De-identified cerebrospinal fluid and plasma samples were obtained from the Cleveland Clinic Lou Ruvo Center for Brain Health Aging and Neurodegeneration Biobank (CBH-biobank) which is approved by the Cleveland Clinic Institutional Review Board for sharing human biospecimens for research purposes. This study was reviewed and approved by the Case Western Reserve University Institutional Review Board for utilization of de-identified human biospecimens. CBH-biobank research participants undergo clinical evaluation, including neurological examination and neuropsychological testing, as well as collection of blood and cerebrospinal fluid for research purposes. The CBH-biobank cerebrospinal fluid and plasma samples obtained for our study included; 20 cognitively normal older adults, 20 mildly cognitively impaired adults, 19 Alzheimer’s disease patients, 20 Parkinson disease patients and 1 Frontal Temporal Dementia patient for a total of 80 unique participant samples. Altogether, 160 samples were tested for asprosin levels. Samples where patients had diabetes or hypertension as potential confounders were removed from the final analysis.

### Viral Vectors for Asprosin Overexpression

To assess the effects of sustained asprosin elevation, six-month-old C57BL/6J 5xFAD or APP^NLG-F^ mice received intravenous tail-vein injections of adeno-associated virus serotype 8 (AAV8) dissolved in 150 μL USP-grade sterile saline. Control mice received AAV8-empty vector (1 × 1012 genome copies (GC)/mouse), whereas experimental mice received AAV8-Asprosin (1 × 10^12^ GC/mouse), encoding N-terminal His-tagged human asprosin preceded by an IL2 signal peptide and driven by the EF1α promoter.

AAV8-mediated transgene expression is known to reach stable levels within approximately 2–3 months following systemic delivery and our specific AAV8 has been widely tested^11,14,15,36,37^.

Consistent with effective elevation of circulating asprosin, AAV8-Asprosin treated mice exhibited sustained increases in food intake and body weight relative to control-treated animals, which served as functional indicators of hormone overexpression. For behavioral studies, mice were injected at 2 or 6 months of age and assessed following sufficient time for stable transgene expression (~ 3 months), as specified for each experiment. For systemic physiology and frailty assessments, six-month-old 5xFAD mice were injected and evaluated at 12 months of age.

### Assessment of Health, Frailty and Sarcopenia

To assess the role of Asprosin-AAV on health deterioration, six-month old 5xFAD were injected intravenously via tail-vein with adeno-associated virus, serotype 8 (AAV8) dissolved in 150 μl USP-grade sterile saline and assessed at 12 months of age.

Measurements of body mass, food and water intake, 24-h urine volume, plasma osmolality, forelimb grip strength, treadmill endurance, wire hang performance, a clinical frailty index, white adipose tissue mass (perigonadal depot) and hindlimb muscle masses (gastrocnemius, soleus and tibialis anterior) were performed. Measurements were performed during the light phase. Animals were acclimated to handling and apparatus for ≥2 sessions before testing to reduce stress-related variability.

#### Food Intake and Body Weight

Food intake and body weight were measured consistently across all experiments. Food intake was assessed using a dustless diet in singly housed mice. To allow habituation, dustless diet pellets (Bio-Ser F0173) were provided for two days before the start of the evaluation. At the beginning of the measurement period, a fresh pre-weighed portion of food was placed on top of each cage in the morning. Food intake was determined by recording the initial food weight and weighing the remaining food 24 hours later.

Mice were weighed individually at the same time each day for any given experiment to minimize variability due to circadian fluctuations in body weight. Each mouse was gently placed in a small container on the balance, allowing for stable and reliable measurements. After weighing, mice were promptly returned to their home cages. The body weight data were recorded and monitored over time to assess changes in response to experimental conditions

#### Water Intake

For manual measurement of water intake, mice were acclimated to sipper water bottles for up to 4 days in solitary housing. After acclimation, 24 hour water intake was measured.

#### 24 hour Urine Volume

For urine volume, mice were subjected to 24 h housing in metabolic cages under fasting conditions, with ad libitum access to water. Urine was collected through a filter to separate from feces and at the end of 24 hours the final volume was measured.

#### Plasma Osmolality

The Texas A&M Rodent Preclinical Phenotyping Core was used to determine plasma osmolality. Plasma samples were centrifuged at 17,000*g* for 2 min at room temperature (20–25 °C). Osmolality was measured using an OsmoPRO Multi-Sample Micro-Osmometer (Advanced Instruments). Two samples were removed due to quality.

#### Forelimb Grip Strength

Forelimb strength was measured using a calibrated digital grip strength meter (Bioseb) with a horizontal metal grid. Mice were held by the base of the tail and allowed to grasp the grid with both forepaws; they were then pulled steadily backwards along the meter’s axis until release. Three trials per mouse were performed with 10 minute inter-trial intervals and averaged. Data analysis was performed under blinded conditions

#### Treadmill

Endurance performance was assessed using a motorized treadmill set to a fixed 15° incline. Mice were acclimated to the treadmill environment prior to testing. On the test day, each mouse was placed on the belt and run at a constant speed of 20 cm/s until exhaustion, defined by failure to resume running (demonstrated by paw lifting) after three consecutive gentle prods or refusal to continue despite mild encouragement. Time to exhaustion and total distance run were recorded for each animal as indices of endurance capacity. Data analysis was performed under blinded conditions

#### Wire Hang

Neuromuscular coordination and fatigue resistance were evaluated using a standardized wire-hang assay. Mice were placed on a horizontal metal wire and allowed to grasp with their forepaws. The latency to fall was measured over a maximum of 180 s. Each animal completed two independent trials separated by at least 15 min, and the mean latency across trials was used for analysis. Data analysis was performed under blinded conditions

#### Frailty Score:

Frailty was quantified using a mouse clinical frailty index adapted from established protocols^56^. Thirty-one health deficits across integument, musculoskeletal, ocular, nasal/oral, urogenital, digestive, respiratory, and neurologic systems were assessed by a trained observer blinded to the group. Each item was scored 0 (no deficit), 0.5 (mild deficit) or 1 (severe deficit) based on predefined criteria (for example: coat condition, piloerection, kyphosis, body condition score, tremor, gait, hearing startle, ocular discharge, alopecia, tail stiffness, menace response, menace startle, whisker loss). The frailty score for each mouse was calculated as the sum of their values on all 31 parameters. Data analysis was performed under blinded conditions One mouse (5xFAD;AAV8-Asprosin) was not evaluated due to recent fighting injuries. One mouse died before frailty score and tissue was collected (5xFAD;AAV8-Empty)

#### White Adipose

Perigonadal white adipose tissue (WAT) was collected bilaterally at necropsy following anesthetic overdose and cervical dislocation. Fat pads were dissected free of associated vessels and connective tissue, washed in PBS, blotted dry, and weighed immediately on an analytical balance. Left and right pad masses were averaged to yield perigonadal WAT mass (g) per mouse.

#### Muscle weight

Hindlimb muscles (gastrocnemius, soleus and tibialis anterior) were dissected bilaterally according to standard anatomical landmarks, trimmed of tendons and visible connective tissue, blotted and weighed immediately. For each muscle, left and right masses were averaged to obtain mean muscle mass (g).

### Behavioral Assays

For all behavioral assays, mice exhibiting fight-related injuries or deemed unwell by ARC veterinary staff were excluded from the study. 5xFAD mice were injected with AAV and tested by Bijoya Basu at Case Western Reserve University, and APP^NL-G-F^ mice were injected with AAV and tested at Baylor College of Medicine by Hesong Liu.

#### Open Field:

The open field assay was used to evaluate general motor activity and anxiety-like behavior, following established protocols. Mice were tested in a 4-arena (50 cm × 50 cm) system (ANYmaze, Stoelting) for 15 minutes, with their order randomized. The apparatus was cleaned with 70% ethanol between trials to minimize olfactory cues. Data analysis was performed under blinded conditions. Open field locomotor activity (distance traveled, mean speed) was assessed in this cohort and is also used as a motor control in a companion manuscript focusing on anxiety-like behavior (Basu et al., title under review). The same open field dataset is used in both studies solely to document the absence of motor deficits for the respective behavioral assays.

#### Novel Object Recognition:

The same apparatus as the open field assay was used for the novel object recognition (NOR) test to assess recognition memory. Testing occurred the day after the open field assay. On Day 1, mice were exposed to two identical objects (50 mL Falcon tubes). On Day 2, one object was replaced with a novel object (three 15 mL Falcon tubes attached together). Interaction times with each object on Day 2 were recorded in a blinded manner. The recognition index was calculated as Recognition Index = 100 × (novel object interaction time / total interaction time). The apparatus was cleaned with 70% ethanol between trials.

#### Barnes Maze:

The Barnes maze consisted of a 92 cm diameter circular platform with 20 evenly spaced 5 cm holes, one of which served as the target escape hole under an escape box. The maze was elevated 75 cm above the floor, with uniform illumination at 800 lux. During habituation, each mouse was placed in the maze center under a glass cylinder for 30 seconds before being guided to the escape box, where they explored for 2 minutes and then stayed for 1 minute. Training consisted of three trials on Day 1 and two trials on Day 2. Mice started under a covered box in the maze center and were allowed 2 minutes to find the escape hole. If successful, mice stayed in the escape box for 1 minute; if unsuccessful, they were guided to it. Visual cues around the room aided spatial orientation. A probe trial was conducted 48 hours after training to assess spatial memory. The escape box was removed, and mice explored for 120 seconds. Time spent in the target quadrant and the number of holes searched were recorded. Data analysis was performed under blinded conditions.

#### Fear Conditioning:

Fear conditioning was performed using conditioning chambers (20 cm × 20 cm × 30 cm; Med Associates) with three metal walls, a transparent front, a grid floor for foot shocks, and a speaker for auditory cues. Chambers were cleaned with 70% ethanol between subjects.

On Day 1, mice were acclimated to the chamber for 3 minutes. During the next 30 seconds, a conditioned stimulus (CS; 5000 Hz tone, 80 dB) was presented, co-terminating with an unconditioned stimulus (US; 0.5 mA foot shock, 1 second). This pairing was repeated four times with 60-second inter-trial intervals. Mice were returned to their home cages 60 seconds after the last shock.

On Day 2, cued fear memory was assessed. For cued, mice were placed in a novel context (different chamber with distinct visual and olfactory cues) for 3 minutes to acclimate. Subsequently, the auditory CS tone was presented twice for two 30 second periods with an inter-trial interval of 60 seconds. Freezing during the CS presentation was recorded and averaged.

All fear conditioning data were collected under blinded conditions. 5xFAD mice were tested on Med Associates fear Conditioning Apparatus, base dimensions: 20cm × 20cm. Apparatus was cleaned with 70% ethanol in between animals. APP^NL-G-F^ mice were tested on Fusion Stimulus Hub with SuperFlex Open Field system (OmniTech Electronics, Inc), base dimensions: 60cm X 60cm. The chambers were cleaned with soapy water between animals.

### AP Staining to Assess Asprosin Binding

Human asprosin was fused with secreted alkaline phosphatase (AP) in the pAPtag-5 vector (APTAG Kit B, GenHunter Corporation; Q202). 293T cells were cultured in 10 cm dishes and transfected with 15 μg of pAPtag-5-Asprosin using FuGENE HD (Promega E2311), following the manufacturer’s protocol. Sixteen hours post-transfection, the media was replaced with serum-free DMEM. AP-tagged asprosin was secreted into the media and collected over a period of four days. The media was then concentrated to less than 500 μl using 10Kda Amicon centrifugal filters. Brains from adult WT C57BL/6 mice were dissected, embedded in OCT, and coronally sectioned. Frozen sections were washed with HBS buffer and subsequently rinsed with HBHA buffer (GenHunter). AP-tagged asprosin protein was applied to the slides and incubated for 90 minutes at room temperature (20–25 °C) in a humidified chamber. Following incubation, sections were washed with HBHA buffer and fixed for 15 seconds using an acetone-formaldehyde fixative. The sections were then washed twice with HBS before being incubated in HBS at 65 °C for 15 minutes to inactivate endogenous phosphatases. Finally, sections were stained with AP assay reagent S (GenHunter) at room temperature, and the reaction was halted using PBS-10 mM EDTA.

### Asprosin ELISA

A custom-built sandwich ELISA was employed to measure plasma asprosin levels in both humans and mice with Alzheimer′s disease, as well as in their respective control groups. For this assay, 25 μL of plasma or 50 μL of CSF was used, with asprosin captured by a fully human anti-asprosin monoclonal antibody, which was developed from a naïve human phage display antibody library by panning against recombinant full-length human asprosin (Texas Therapeutics Institute at the University of Texas Health Science Center at Houston). A mouse anti-asprosin monoclonal antibody, targeting human asprosin amino acids 106–134 (corresponding to human profibrillin amino acids 2838–2865), served as the detection antibody. An HRP-linked anti-mouse secondary antibody was used to generate the detection signal. To create a standard curve, recombinant mouse asprosin produced in mammalian cells (AdipoGen AG-40B-0174T-C010) was used. The blocking, coating, substrate, and stop solutions were purchased from SeraCare.

### Western Blot Analysis

Snap-frozen cerebellar tissue from both WT and 5xFAD mice, as well as from humans with Alzheimer’s disease (AD) and unaffected controls, was homogenized using N-PER Neuronal Protein Extraction Reagent (Thermo Fisher Scientific, 87792) supplemented with protease inhibitor (Thermo Fisher Scientific, 78429) and phosphatase inhibitor (Thermo Fisher Scientific, 78420) to produce protein lysates. Protein concentrations were quantified using the Pierce BCA Protein Assay Kit (Thermo Fisher Scientific, 23227).

Subsequently, 25 μg of protein lysate was loaded onto a 4–12% Bolt Bis-Tris Protein Gel (Thermo Fisher Scientific, NW04127BOX) and electrophoresed for approximately 1.5 hours using Bis-Tris running buffer. Precision Plus Protein Kaleidoscope (Bio-Rad, 1610375) was used as the molecular weight ladder. Proteins were transferred onto nitrocellulose membranes using the Invitrogen Power Blotter System for 10 minutes at room temperature.

Membranes were blocked with Clear Milk Blocking Buffer (Thermo Fisher Scientific, 37587), diluted 1X in TBST, for 1 hour at room temperature. Primary antibodies were diluted in the same blocking buffer and incubated with the membranes overnight at 4°C. Following primary antibody incubation, membranes were washed with 1X TBST and then incubated with HRP-conjugated mouse or rabbit secondary antibodies, diluted in 1X TBST, for 2 hours at room temperature. Chemiluminescent signals were detected using a 1:1 ratio of HRP substrates (Thermo Fisher Scientific, 34577 and 34094).

For all western blots, molecular weight markers and experimental samples were run on the same gel. Membranes were cut at approximately 50 kDa, allowing separate incubation of the segments with antibodies against PTPRD and β-actin. Primary antibodies used were Rabbit Polyclonal anti-PTPRD (1:500; ABclonal, A15713) and Mouse Monoclonal anti-β-actin (1:2000; Cell Signaling, 8H10D10). Secondary antibodies included HRP-conjugated anti-rabbit IgG (1:10,000; Cytiva, NA934) and HRP-conjugated anti-mouse IgG (1:10,000; GeneTex, GTX213112–01).

### *In vivo* assessment of phospho-Stat3

Age and sex matched de-identified human donor samples of Alzheimer′s disease and healthy controls from cerebellum and hippocampus were obtained. Cerebellar and hippocampal tissue was homogenized using N-PER Neuronal Protein Extraction Reagent (Thermo Fisher Scientific, 87792) supplemented with protease inhibitor (Thermo Fisher Scientific, 78429) and phosphatase inhibitor (Thermo Fisher Scientific, 78420) to produce protein lysates. Protein concentrations were quantified using the Pierce BCA Protein Assay Kit (Thermo Fisher Scientific, 23227). Samples were later subjected to phosphor-Stat3 quantification by ELISA (Abcam; ab126458) and OD values were divided by concentration to obtain final values.

For hippocampal tissue, 9 month old 5xFAD mice were under deep isoflorane anesthesia before cervical dislocation and decapitation. Hippocampus was excised, snap-frozen, and later subjected to phospho-Stat3 quantification by ELISA (Abcam; ab126458) and OD values were divided by concentration to obtain final values.

### Immunohistochemistry Staining:

Mice were deeply anesthetized with a ketamine-xylazine cocktail, confirmed by a toe pinch, and transcardially perfused with 30 mL of 1x phosphate-buffered saline (PBS) followed by 30 mL of 4% paraformaldehyde (PFA) in PBS. Brains were extracted, post-fixed in 4% PFA for 24 hours at 4°C, and cryoprotected in 20% sucrose in PBS.

Brains were sectioned into 30-μm-thick coronal slices using a Leica SM2010 R Sliding Microtome. Sections were stored in cryoprotectant solution (30% ethylene glycol, 20% glycerol in PBS) at −20°C until further use. Prior to staining, sections were rinsed in PBS to remove cryoprotectant, treated with 0.3% H_2_O_2_ in PBS for 30 minutes, washed, and blocked in 10% Normal Donkey Serum (Jackson ImmunoResearch, AB–2337258) for 1 hour.

Sections were incubated overnight at 4°C with primary antibodies diluted in 1% Normal Donkey Serum: Rabbit anti-p-Stat3 (1:1000; Cell Signaling, 9145L) and Monoclonal anti-calbindin (1:500; Sigma-Aldrich, C9848–100UL). The next day, sections were washed and exposed to secondary antibodies (1:200 dilution): goat anti-mouse Alexa 488 (Thermo Fisher, A32723) and goat anti-rabbit Alexa 594 (Thermo Fisher, A32740). Following PBS washes, sections were stained with 300 nM DAPI in PBS, rinsed, mounted onto slides, and sealed with Vectashield mounting medium (H-1000).

Fluorescently labeled sections were imaged using a Zeiss Axio Scan.Z1 slide scanner with consistent acquisition settings across all samples for quantitative analysis.

### Immunohistochemistry Analysis:

Similar Regions of Interest for analysis and quantification were selected using image processing software (ImageJ). Cerebellar regions were cropped and analyzed for simultaneous expression of phosphorylated Stat3 (red color channel) and Calbindin (green color channel). In the cerebellum, calbindin only stains Purkinje neurons^57^. Each image was filtered to remove noise using non-local means filtering. Pixels containing expression of phosphorylated Stat3 and calbindin were determined through Otsu thresholding^58^. This isolated the cells and removed the background fluorescence present in the images. The overlap of expression between the two channels was determined by counting the number of pixels that passed the Otsu threshold in both channels. The sum total of these pixels represents the total overlap between the two channels within cells. Overlap was calculated by dividing the total number of pixels by the size of the image, generating a measure for expression per unit area.

### Stereotaxic Injections

Stereotaxic injections were performed on adult *Ptprd*^*Flox/Flox*^ male mice (8–12 weeks old) under isoflurane anesthesia (1.5–2% in oxygen). Mice were placed in a stereotaxic frame (RWD Instruments, China), and an incision was made to expose the skull. The skull was leveled, and stereotaxic coordinates were used to target the ventral hippocampus (AP −3.4 mm, ML ±3.0 mm, and DV −4.5 mm). A small hole was drilled through the skull at the target sites, and a 5 μL microsyringe (Hamilton, USA) was used to deliver 0.15 μL of viral vector (AAV8-Cre-GFP, titer 4×10^12^ vg/mL) at a rate of 0.1 μL/min. Following the injection, the pipette was left in place for 5 minutes to allow diffusion and prevent backflow. Afterward, the incision was sutured, and mice were allowed to recover on a heating pad. Post-operative care included monitoring for pain and infection. Successful targeting was confirmed via histological analysis of GFP expression. Behavioral assays were conducted at least 6 weeks post-op.

#### Immunohistochemistry of Stereotaxic Injection Mice

To confirm deletion of Ptptrd, mice were anesthetized with inhaled isoflurane and perfused with saline, followed by 10% formalin. Brain sections (25 μm in thickness) were collected and then subjected to immunofluorescence for Ptprd as described previously^36^. Briefly, brain sections were blocked (5% normal donkey in 0.1% PBST) for 1 hour. Then, the brain sections were incubated overnight with rabbit anti-Ptprd antibody (1:200 dilution; #A15713, ABclonal) on a shaker at 4°C overnight. The next day, the brain sections were incubated with the donkey anti-rabbit Alexa Fluor 594 (1:500; A21206, Invitrogen) for 2 hours. Sections were mounted on slides and coverslipped with 4′,6-diamidino-2-phenylindole mounting medium. Fluorescence images were taken using the Nikon Eclipse FN1 fluorescence microscope with ORCA-Fusion digital camera C14440 (HAMAMTSU, Japan). Data from four different mice were used in statistical analyses.

#### Electrophysiology readings

*Ptprd*^*Flox/Flox*^ and WT mice received AAV-CamKII-EGFP-WGA in ventral brain region were used for electrophysiology recordings. On the day of the electrophysiology recording experiment, mice were euthanized under fed or fasted conditions. Then, the entire brains of the mice were removed and immediately submerged in ice-cold sucrose-based cutting solution (adjusted to pH 7.3) containing (in mM): 10 NaCl, 25 NaHCO3, 195 Sucrose, 5 Glucose, 2.5 KCl, 1.25 NaH_2_PO_4_, 2 Na pyruvate, 0.5 CaCl_2_, 7 MgCl_2_ bubbled continuously with 95% O2 and 5% CO2^1–3^. The slices (250 μm) were cut with a VT1200 S vibratome (Leica) and recovered for 1 h at 34°C and then maintained at room temperature in artificial cerebrospinal fluid (aCSF, pH 7.3) containing: 126 mM NaCl, 2.5 mM KCl, 2.4 mM CaCl2, 1.2 mM NaH_2_PO_4_, 1.2 mM MgCl_2_, 11.1 mM glucose, and 21.4 mM NaHCO_3_ saturated with 95% O_2_ and 5% CO_2_. TOMATO(+) neurons were visualized using epifluorescence and IR-DIC imaging on an upright microscope equipped with a moveable stage (MP-285, Sutter Instrument).

For electrophysiological recording, brain slices were superfused at 34°C in oxygenated aCSF at a flow rate of 1.8–2 ml/min as described previously^4^. Patch pipettes with resistances of 3–5 MΩ were filled with intracellular solution (pH 7.3) containing: 128 mM K-Gluconate, 10 mM KCl, 10 mM HEPES, 0.1 mM EGTA, 2 mM MgCl_2_, 0.05 mM Na-GTP and 0.05 mM Mg-ATP. Recordings were made using a MultiClamp 700B amplifier (Axon Instrument), sampled using Digidata 1440A, and analyzed offline with pClamp 10.3 software (Axon Instruments). Series resistance was monitored during the recording, and the values were generally <10 MΩ and were not compensated. Data were excluded if the series resistance increased dramatically during the experiment or without overshoot for the action potential. Currents were amplified, filtered at 1 kHz, and digitized at 20 kHz. The current clamp was engaged to test neural firing frequency and resting membrane potential (RM) in control and Ptprd^vHippocampus^ KO neurons after asprosin treatment (1s puff, 30 nM). The values for RM and firing frequency averaged within the 2-min bin. Action potential frequency and resting membrane potential were measured using the Mini Analysis program (Synaptosoft Inc.).

A voltage-clamp protocol was used to record the SK current as we did before^36^. Briefly, patch pipettes with resistances of 3–5 MΩ were filled with intracellular solution (pH 7.3) containing 128 mM K-Gluconate, 10 mM KCl, 10 mM HEPES, 0.1 mM EGTA, 2 mM MgCl2, 0.05 mM Na-GTP and 0.05 mM Mg-ATP. The SK tail current was evoked by a 100 ms depolarizing pulse from a holding potential of −60 mV to 0 mV and then back to −60 mV with or without asprosin treatment (1s puff, 30 nM).

### Co-Immunoprecipitation

Cerebellar and hippocampal tissue from wildtype and 5xFAD mice were dissected, weighed, and placed in between 500–1000uL of an IP lysis buffer cocktail. Protease and phosphatase inhibitors were added at 10uL per 1mL buffer each. Samples were incubated on ice for 5 minutes following homogenization every 5 minutes for a total of 20 minutes. Both tissues were centrifuged at 14000rpm for 15 minutes in 4C to collect tissue. Pierce^™^ BCA Protein Assay kit (Thermo Fisher; 23227) was utilized to obtain the total protein concentration for each sample.

The Pierce^™^ Crosslink IP kit (Thermo Fisher; 26147) was used to conduct a co-immunoprecipitation reaction. Antibodies were first bound to Pierce Protein A/G Plus Agarose resin (Thermo Fisher; 1861760) through creating a slurry of 5ug of amyloid beta (Invitrogen; 715800), 20X Coupling Buffer (Thermofisher; 1861611) and filtered water to obtain a total volume of 100 uL. Samples were rotated at room temperature for 1 hour. A DSS solution was created by dissolving DSS (Thermo Fisher; 1863418) in DMSO (Sigma Aldrich; D2438–10ML) to make 2.5 mM of DSS to cross-link with the antibody-bound resin. The cross-linking reaction was incubated on a rotator at room temperature for 1 hour followed by quenching the reaction with Elution Buffer (Thermo Fisher; 1858606) and washing with cold IP Lysis/Wash Buffer (Thermo Fisher; 1861603). 1mg protein lysate was incubated with Control Agarose Resin slurry (Thermo Fisher; 1859011) for 45 minutes at 4C on a rotator to pre-clear the lysate. The immunoprecipitation reaction was carried out by incubating 1mg of lysate with the antibody-crosslinked resin for 3 hours at 4C on a rotator. The samples were washed with IP Lysis/Wash Buffer and 1X Conditioning Buffer (Thermo Fisher; 1861612) followed by incubating for 5 minutes in Elution Buffer to quench the reaction.

20uL of IP product was mixed with 5uL of 5X Lane Marker Sample Buffer to make a 1X solution (Thermo Fisher; 1859594). Western blot analysis was conducted to assess receptor-ligand interaction. Each protein sample was heated at 95C for 5 minutes to denature protein. Samples were then loaded and run on NuPAGE^™^ 3–8% Nu-Page, Tris Acetate gel (Thermo Fisher). Invitrogen Power Blotter was used for 10 minutes at room temperature to transfer proteins to nitrocellulose membranes. The membrane was cut in half at 37 kda and blocked separately in Clear Milk Blocking Buffer (in 1XTBST) at room temperature for 45 minutes followed by incubation overnight at 4C in respective primary antibodies (rabbit polyclonal anti-beta Amyloid (1:1000; Invitrogen; 71–5800)), rabbit polyclonal anti-PTPRD (1:500; ABclonal, A15713)) corresponding to the molecular weight of the proteins probed for. On the following day, membranes were incubated with HR-labelled secondary antibody (HRP-conjugated anti-rabbit igG (1:10,000; ECL)) for 1 hour at room temperature and detected using chemiluminescent substrates (Thermo Fisher Scientific, 34577, 34094).

### Cell Culture and Luciferase Assays:

HEK293T cells were cultured at 37°C in 5% CO_2_ in DMEM supplemented with 10% fetal bovine serum (FBS; HyClone) and 100 μg/ml penicillin-streptomycin. For all experiments, HEK293T cells were transfected with the 4xM67 pTATA-TK-Luc plasmid (Addgene, 8688), which contains Stat3-response elements driving luciferase expression.

To evaluate the effects of amyloid β (Aβ) on PTPRD activity, HEK293T cells seeded at 10,000 cells/well in 96-well plates were transfected with 2 μg of the 4xM67 pTATA-TK-Luc plasmid. The following day, 200 nM of recombinant Aβ_1–40_ (Sigma Aldrich, A1075), Aβ_1–42_ Sigma Aldrich, PP69), reverse-sequence Aβ_40–1_ (Sigma Aldrich, A2326), reverse-sequence Aβ_42–1_ (Sigma Aldrich, SCP0048), or recombinant green fluorescent protein (GFP; USBiological, G8965–10E) was added to the cells. After 24 hours, Stat3-mediated luciferase activity was measured.

To investigate competition between Aβ and asprosin, HEK293T cells transfected with 2 μg of the 4xM67 pTATA-TK-Luc plasmid were transfected with 400 ng of either an asprosin plasmid expressing N-terminal His-tagged human asprosin preceded by an IL2 signal peptide and driven by the EF1α promoter or an empty vector control. Twenty-four hours post-transfection, 200 nM Aβ_1–40_, Aβ_1–42,_ Aβ_40–1,_ Aβ_42–1_ or GFP was added in fresh media. After 6 hours, Stat3-driven luciferase activity was measured.

To assess the effect of PTPRD loss on Stat3 activity, HEK293T cells transfected with 2 μg of the 4xM67 pTATA-TK-Luc plasmid were transfected with either 25 nM PTPRD-specific siRNA or pooled scrambled siRNA (Dharmacon, T-2001–02) using the manufacturer’s protocol. Twenty-four hours after siRNA transfection, cells were treated with 200 nM Aβ_1–40_, Aβ_1–42,_ Aβ_40–1,_ Aβ_42–1_ or GFP. Forty-eight hours post-transfection (24 hours after protein treatment), cells were lysed to assess siRNA knockdown efficiency, measure phospho-Stat3 levels via western blot, and quantify Stat3-mediated luciferase activity.

Transfection of HEK293T cells was performed using FuGENE HD transfection reagent (Promega, E2312) according to the manufacturer’s instructions. Cells were lysed using Reporter Lysis 5X Buffer (Promega, E3971), and luciferase activity was measured using Luciferase Assay Reagent (Promega, E1483). All assays were performed following standard manufacturer protocols. For cell-based assays, a biological replicate was defined as an independent well of cells subjected to separate transfection and treatment conditions, whereas technical replicates represent repeated measurements within the same experiment. Experiments were repeated across multiple independent culture preparations with consistent results.

### Surface plasmon resonance (SPR):

Surface plasmon resonance (SPR) studies were performed using Biacore T200 (Cytivia) with PTPRD extracellular domain (ECD) (Acro Biosystems, PTD-H52H9) covalently immobilized on an S series CM5 sensor chip via amine coupling. Recombinant human amyloid-β_1–42_ peptides (Sigma Aldrich AG968–1MG) were initially dissolved in hexafluoroisopropanol (HFIP) to disaggregate pre-existing assemblies, dried under vacuum, and resuspended in PBSP+ (Cytivia) running buffer immediately prior to analysis. Preparations were vortexed for 10 minutes before injection. Aβ peptides were injected over immobilized PTPRD at serial concentrations (1000nM, 500 nM, 250 nM, 125 nM, 62.5 nM, 31.25 nM, 15.625 nM) at 25 °C with a flow rate of 30 μL/min.

For competition experiments, recombinant human asprosin concentrations from 18.75nM to 300nM in series dilution were co-injected with 25nM of Aβ at 30 μL/min over the Ptprd bound sensor chip at 25 C to assess competitive binding. Surface was regenerated with Glycine, pH 1.5 for 30s at 30 μL/min. Two different recombinant asprosin preparations were tested (Novus Biologicals NBP3–18164; Biobyrt, orb1784787). Binding data were analyzed using BIAevaluation software (Cytiva) and replotted using Origin software.

### Quantification and Statistical Analysis

Data were graphed and analyzed using GraphPad Prism (version 9 or higher). Sample sizes and statistical approaches were determined *a priori* based on experimental design and preliminary data. Sample exclusions were predefined and are reported explicitly in the Methods. Results are reported as mean ± standard error of the mean (SEM), with individual data points displayed where appropriate. Comparisons between two groups were performed using unpaired Student’s *t*-tests, whereas analyses involving more than two groups were conducted using one-way or two-way analysis of variance (ANOVA) followed by Bonferroni post hoc correction for multiple comparisons. For experiments involving repeated measurements of the same variable, repeatedmeasures ANOVA was employed.

For select analyses of phospho-STAT3 levels, one-sided statistical tests were used based on *a priori* directional hypotheses derived from established Ptprd signaling biology, in which reduced Ptprd activity is predicted to increase STAT3 phosphorylation. These analyses were prespecified before data collection and were applied consistently across experimental groups. All other statistical tests were two-sided, as indicated. Sex was treated as a biological variable throughout the study. Where both sexes were included, animals were age- and sex-matched across experimental groups, and analyses were performed within sex-matched cohorts or separately by sex, as indicated in the figure legends. AD and control subjects were age- and sex-matched at the cohort level. Because samples were obtained from different individuals, observations were treated as independent and group differences were assessed using unpaired statistical tests. The study was not powered to detect sex-specific differences unless explicitly stated. Animals were randomly assigned to experimental or treatment groups and the order mice were tested in any given day for behavioral assays was randomized. All behavioral testing, immunohistochemical quantification, and image analyses were performed with the experimenter blinded to group allocation. Statistical significance was defined as α = 0.05.

## Supplementary Material

1

## Data Availability

All data generated or analyzed during this study are available in the main text or the supplementary materials. Custom code was used for automated image analysis filtering and overlap calculation between Calbindin and p-Stat3 and has been shared to a public repository (GitHub). Materials used in this study are available from the corresponding authors upon reasonable request, subject to applicable institutional guidelines and material transfer agreements.
