# Supplementary material for: Asprosin–PTPRD endocrine resistance links brain dysfunction and systemic wasting in Alzheimer’s disease
Source: Res Sq. 2026 Jun 17:rs.3.rs-9972826. Preprint. [Version 1] doi: 10.21203/rs.3.rs-9972826/v1 (PMC13308377; doi:10.21203/rs.3.rs-9972826/v1)
Supplement: 1 [file NIHPPRS9972826V1-supplement-1.pdf]

Supplemental Figures

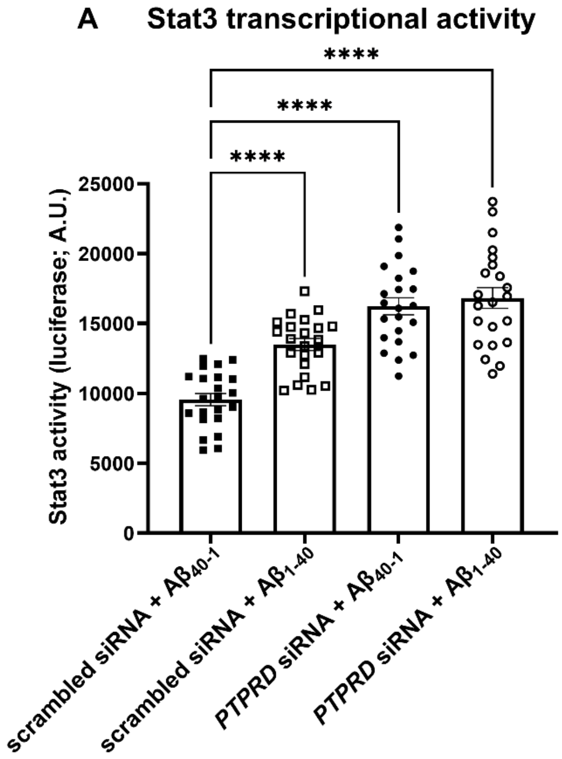

Supplemental Figure 1: Aβ antagonizes Ptprd phosphatase signaling

(A) STAT3 luciferase activity in HEK293T cells transfected with reporter and empty or IL2-his-asprosin plasmid, treated with 200 nM $A\beta_{1-40}$ or $A\beta_{40-1}$ control; measured 6 h after treatment (three technical replicates, 10 biological replicates/group).

Data are mean ± SEM with individual points. One-way ANOVA (A). *$p<0.05$, **$p<0.01$, ***$p<0.001$, ****$p<0.0001$.

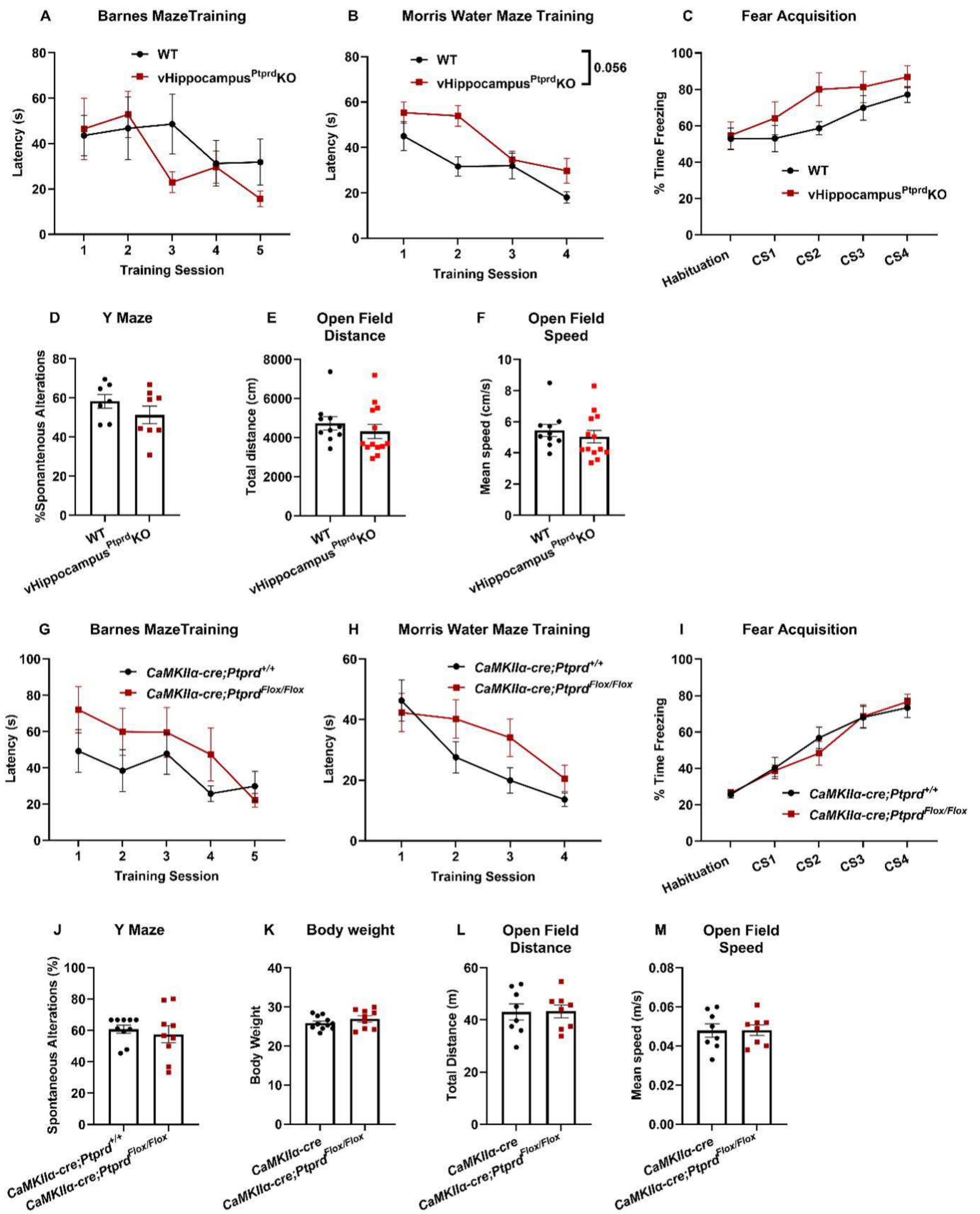

Supplemental Figure 2: Reduced Ptprd activity results in the loss of circuit-specific memory domains

(A–C) Training/acquisition in WT and vHippocampus^{Ptprd} KO mice: Barnes Maze (A; n=6 WT, n=7 KO), Morris Water Maze (B; n=8/group), and Cued Fear Conditioning (C; n=6 WT, n=7 KO). (D–F) Working memory by Y Maze (D; n=7 WT, n=8 KO), Open field distance (E; n=10 WT, n=13 KO), and Open field mean speed (F; n=10 WT, n=13 KO) in vHippocampus^{Ptprd} KO vs WT. (G-I) Training/acquisition in 10–12-week-old male CaMKII-cre;Ptprd$^{Flox/Flox}$ mice vs CaMKII-cre controls: Barnes Maze (G; n=9/group), Morris Water Maze (H; n=7/group), and Cued Fear Conditioning (I; n=9 KO, n=11 control). (J-M) Working memory by Y Maze (J; n=9 KO, n=10 control), Body weight (K; n=9 KO, n=11 control) Open field distance (L; n=8 KO, n=8 control), and Open field mean speed (M; n=8 KO, n=8 control) in CaMKII-cre;Ptprd$^{Flox/Flox}$ vs controls.

Data are mean ± SEM (A–C,G-I), mean ± SEM with individual points (D-F,J-M). Two-way ANOVA (A–C,G-I); two-sided unpaired (D–F,J-M) t-tests. *p<0.05, **p<0.01, ***p<0.001, ****$p<0.0001$.

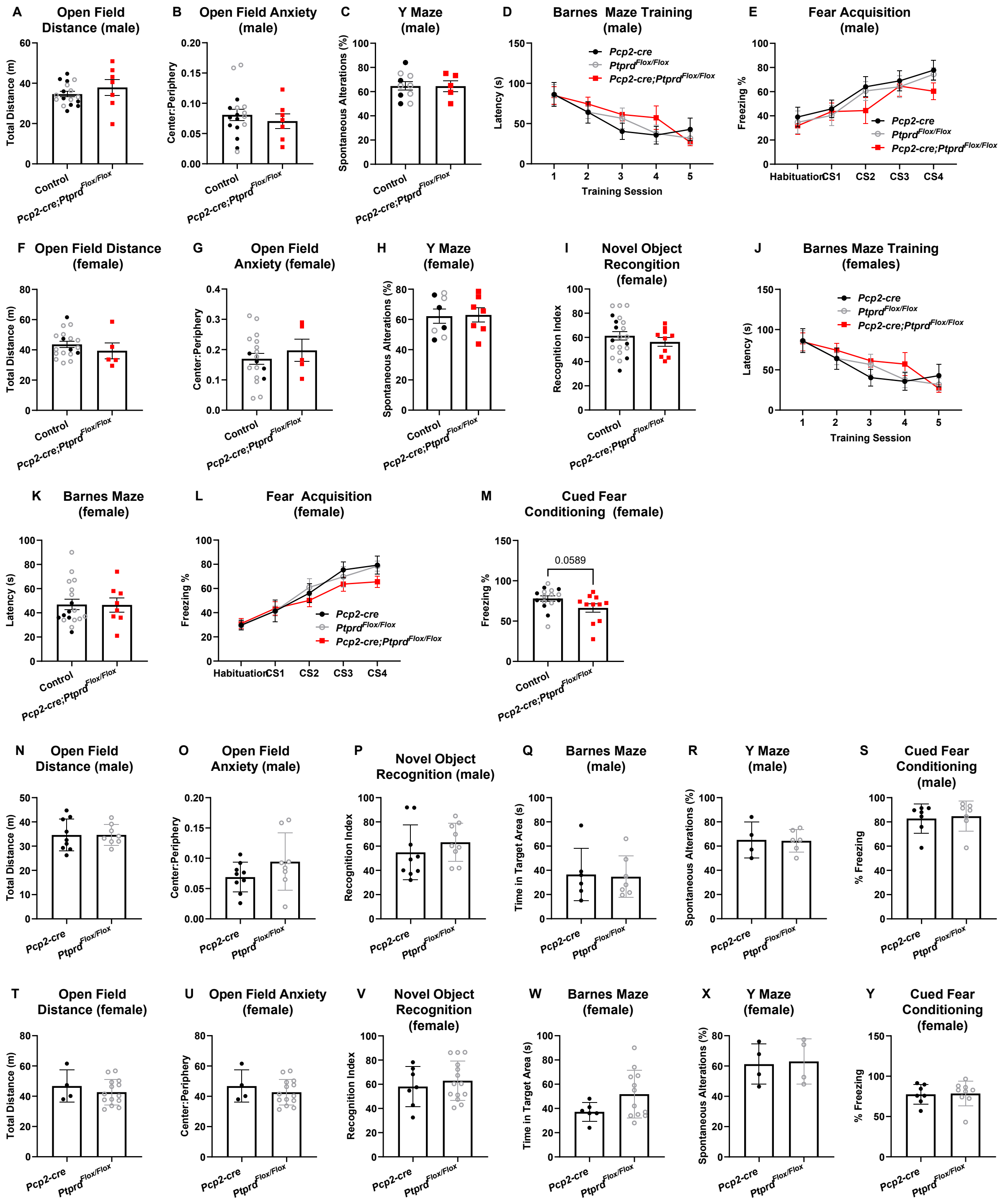

Supplemental Figure 3: The impact of reduced Ptprd activity on circuit-specific memory domains

(A–E) Male Pcp2-cre;Ptprd$^{Flox/Flox}$ mice vs controls: open field total distance (A; n=7 vs n=9, n=8) and center-to-periphery ratio (B; n=7 vs n=9, n=8), Y Maze (C; n=5 vs n=4, n=6), Barnes Maze training (D; n=10 vs n=6, n=7), and fear acquisition (E; n=8 vs n=7, n=7).

(F–M) Female Pcp2-cre;Ptprd$^{Flox/Flox}$ mice vs controls: open field distance (F; n=5 vs n=4, n=14) and ratio (G; n=5 vs n=4, n=14), Y Maze (H; n=7 vs n=4, n=4), Novel Object Recognition (I; n=10 vs n=7, n=14), Barnes Maze training and memory (J,K; n=8 vs n=6, n=12), fear acquisition (L; n=11 vs n=7, n=9), and associative memory (M; n=11 vs n=7, n=9).

(N–S) Male controls (Pcp2-cre vs Ptprd$^{Flox/Flox}$): open field distance (N; n=9 vs n=8) and ratio (O; n=9 vs n=8), Novel Object Recognition (P; n=9/group), Barnes Maze (Q; n=6 vs n=7), Y Maze (R; n=4 vs n=6), and fear conditioning (S; n=7/group).

(T–Y) Female controls: open field distance (T; n=4 vs n=14) and ratio (U; n=4 vs n=14), Novel Object Recognition (V; n=7 vs n=14), Barnes Maze (W; n=6 vs n=12), Y Maze (X; n=7 vs n=9), and fear conditioning (Y; n=7 vs n=9).

Data are mean ± SEM (D,J,L) or mean ± SEM with individual points (A–C,E–H,K,M–Y). Two-way ANOVA (D,J,L); two-sided unpaired t-tests (A–C,E–H,K,M–Y). *p<0.05, **p<0.01, ***p<0.001, ****$p<0.0001$.

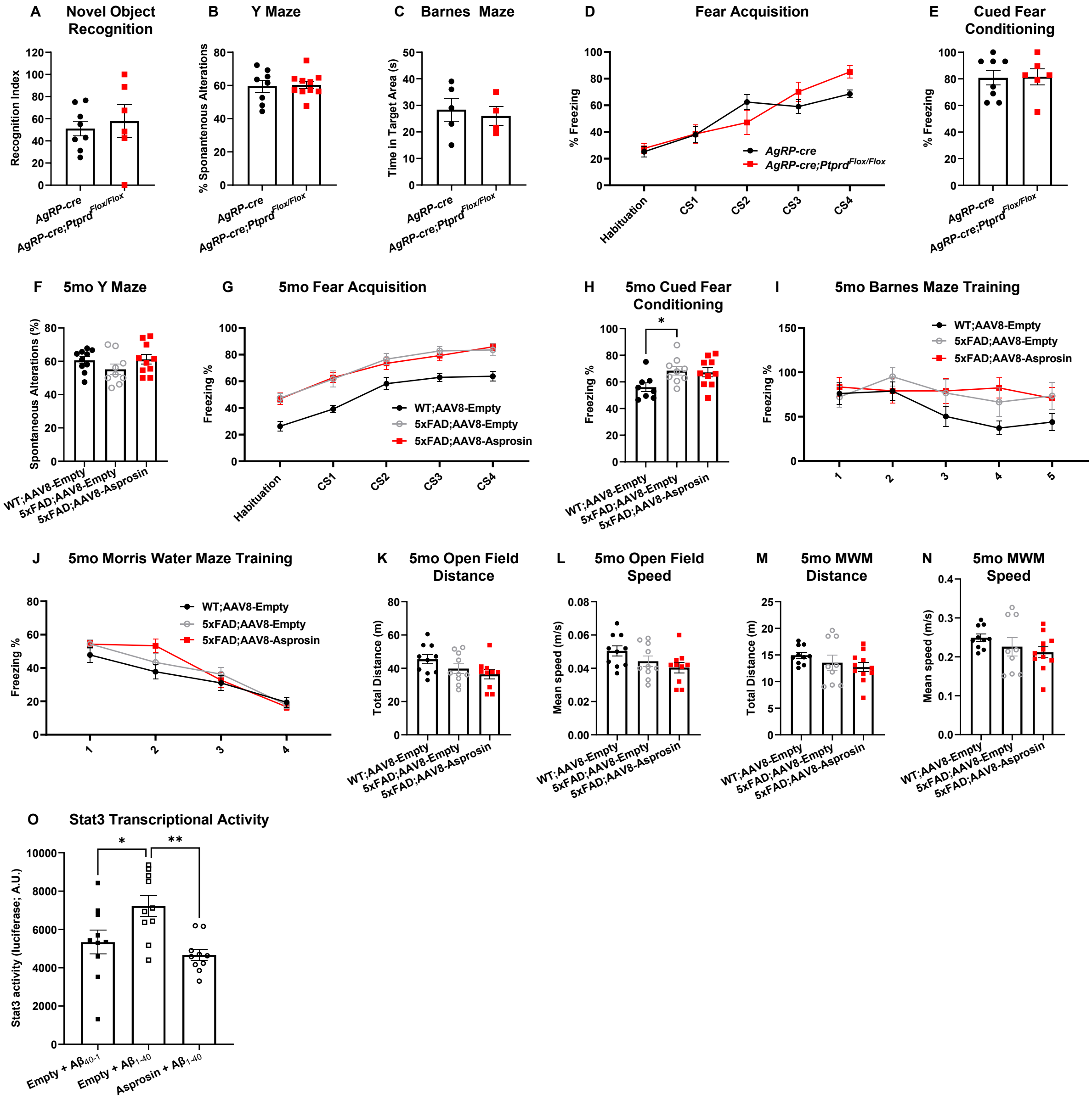

Supplemental Figure 4: The impact of the asprosin-Ptprd axis on receptor signaling and distinct memory domains

(A–E) Behavioral assays in 12-week-old male AgRP-cre;Ptprd$^{Flox/Flox}$ mice vs AgRP-cre controls: Novel Object Recognition (A; n=6 vs n=8), Y Maze (B; n=10 vs n=8), Barnes Maze (C; n=4 vs n=5), fear acquisition (D; n=6 vs n=8), and associative memory (E; n=6 vs n=8). (F–J) Memory in 5-month-old males: WT mice (AAV8-Empty) and 5xFAD mice (AAV8-Empty or AAV8-Asprosin; 1×10^{12} GC/mouse) assessed by Y Maze (F), fear acquisition (G), cue-dependent freezing (H; n=8–10/group), Barnes Maze training (I), and Morris Water Maze training (J).

(K-N) Behavioral assays in 5-month-old males: WT mice (AAV8-Empty) and 5xFAD mice (AAV8-Empty or AAV8-Asprosin; 1×10^{12} GC/mouse), Open field distance (K) , and mean speed (L), Morris Water Maze distance (M), and mean speed (N). (O) STAT3 luciferase activity in HEK293T cells transfected with reporter and empty or IL2-his-asprosin plasmid, treated with 200 nM $A\beta_{1-40}$ or $A\beta_{40-1}$ control; measured 6 h after treatment (three technical replicates, 10 biological replicates/group).

Data are mean ± SEM (D,G,I,J) or mean ± SEM with individual points (A–C,E,F,H,K-O). Two-way ANOVA (D,G,I,J); one-way ANOVA with Tukey post hoc (F,H,O); two-sided unpaired t-tests (A–C,K-N). *p<0.05, **p<0.01, ***p<0.001, ****$p<0.0001$.

.

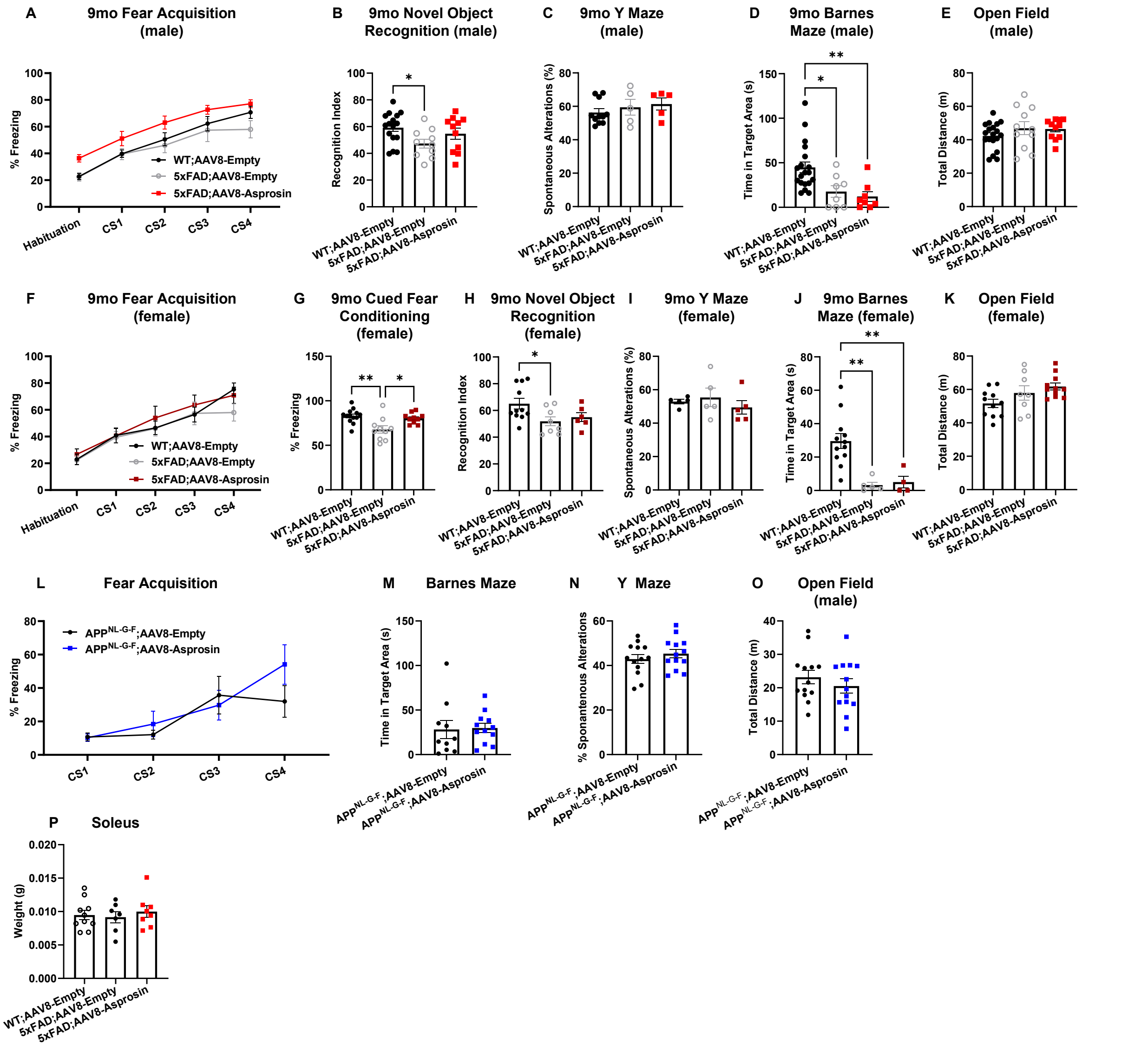

Supplemental Figure 5: Asprosin supplementation in two distinct AD models

(A–D) Memory in 9-month-old males: fear acquisition (A), Novel Object Recognition (B; n=10–16/group), Y Maze (C; n=11 WT, n=5 per 5xFAD group), and Barnes Maze (D; n=19 WT, n=8 per 5xFAD group).

(E) Locomotion in 9-month-old males (n=19 WT, n=11 per 5xFAD group).

(F,G) Associative memory in 9-month-old females: WT (AAV8-Empty) and 5xFAD (AAV8-Empty or AAV8-Asprosin; 1×10^{12} GC/mouse) assessed by fear acquisition (F) and cue freezing

(G; n=10–13/group).

(H–J) Memory in 9-month-old females: Novel Object Recognition (H; n=6–11/group), Y Maze (I; n=5/group), and Barnes Maze (J; n=12 WT, n=4–5 per 5xFAD group).

(K) Locomotion in 9-month-old females (K; n=11 WT, n=8 5xFAD-Empty, n=11 5xFAD-Asprosin).

(L–O) Memory and locomotion in APP^{NL-G-F} mice: fear conditioning (L; n=12/group), Barnes Maze (M; n=10, n=12), Y Maze (N; n=13/group), and open field (O; n=13/group).

(P) Soleus muscle weight in 12-month-old WT (n=10), 5xFAD-Empty (n=7), and 5xFAD-Asprosin (n=8).

Data are mean ± SEM (A,C,L) or mean ± SEM with individual points (B,D–K,M–P). Two-way ANOVA (A,B,L); one-way ANOVA with Tukey post hoc (B,D–K,P); two-sided unpaired t-tests (M–O). *p<0.05, **p<0.01, ***p<0.001, ****$p<0.0001$.
